# Molecular Mechanisms of Inhibition of *Streptococcus* Species by Phytochemicals

**DOI:** 10.3390/molecules21020215

**Published:** 2016-02-17

**Authors:** Soheila Abachi, Song Lee, H. P. Vasantha Rupasinghe

**Affiliations:** 1 Faculty of Agriculture, Dalhousie University, Truro, NS PO Box 550, Canada; sh764873@dal.ca; 2 Faculty of Dentistry, Dalhousie University, Halifax, NS PO Box 15000, Canada; Song.Lee@dal.ca

**Keywords:** streptococci, biofilm, adherence, phytochemical, quorum sensing, *S. mutans*, *S. pyogenes*, *S. agalactiae*, *S. pneumoniae*

## Abstract

This review paper summarizes the antibacterial effects of phytochemicals of various medicinal plants against pathogenic and cariogenic streptococcal species. The information suggests that these phytochemicals have potential as alternatives to the classical antibiotics currently used for the treatment of streptococcal infections. The phytochemicals demonstrate direct bactericidal or bacteriostatic effects, such as: (i) prevention of bacterial adherence to mucosal surfaces of the pharynx, skin, and teeth surface; (ii) inhibition of glycolytic enzymes and pH drop; (iii) reduction of biofilm and plaque formation; and (iv) cell surface hydrophobicity. Collectively, findings from numerous studies suggest that phytochemicals could be used as drugs for elimination of infections with minimal side effects.

## 1. Introduction

The aim of this review is to summarize the current knowledge of the antimicrobial activity of naturally occurring molecules isolated from plants against *Streptococcus* species, focusing on their mechanisms of action. This review will highlight the phytochemicals that could be used as alternatives or enhancements to current antibiotic treatments for *Streptococcus* species. The scope of the review is limited to inhibitory effects of phytochemicals, mainly polyphenols, against *Streptococcus* species and where possible, their mechanisms of action against the major virulence factors will be discussed. Due to their major implications on human health, this review has largely focused on four *Streptococcus* species: (i) *S. mutans* (ii) *S. pyogenes* (iii) *S. agalactiae* and (iv) *S. pneumoniae.* To explain the potential mechanisms of inhibition of the phytochemicals, *S. mutans* has been used as the major example.

### 1.1. Streptococci

*Streptococcus* species are bacteria belonging to the Firmicutes phylum under the order of Lactobacillales and the family of Streptococcaceae [1]. Three genera exist within the family of Streptococcaceae including *Streptococcus*, *Lactococcus* and *Lactovum* of which *Streptococcus* is most diverse, containing 79 species [1]. A number of *Streptococcus* species are pathogenic to humans and animals, with *S. pyogenes* and *S. pneumoniae* as the most important pathogens [1]. These Gram positive bacteria generally appear as pairs or chains, are spherical to ovoid in shape, nutritionally fastidious, with fermentative metabolism and many of them form capsules [2].

*Streptococcus* species are found mostly in the oral cavity and nasopharynx and form a significant portion of the normal microbiota of humans and animals [2,3]. In healthy individuals, normal microbiota are harmless, however, they can cause infection under certain conditions, such as immune compromised stage [2,4]. *Streptococcus* species (e.g., *S. pyogenes*, *S. agalactiae*, and *S. pneumoniae*) can be classified serologically based on the cell wall carbohydrates into groups A to V [2,5,6]. Streptococci can also be grouped based on morphological differences, type of hemolysis on blood agar, biochemical reactions, cell wall pili-associated protein, and polysaccharide capsule (specific for group B streptococci) [7]. To date more than 85 capsule antigenic types of *S. pneumoniae*, 124 serotypes of *S. pyogenes* and nine CPS (capsular polysaccharide) serotypes of *S. agalactiae* have been proposed [7,8,9]. The cell wall of streptococci is among the most studied bacterial cell walls [7,10].

### 1.2. Streptococcal Infections and Major Virulence Factors

The diseases caused by streptococci range from non-life-threatening conditions like dental caries, pharyngitis (strep throat) to life-threatening conditions such as necrotizing fasciitis and meningitis (Table 1) [5]. Of all the oral streptococci, *S. mutans* is considered to be the etiological agent of dental caries. According to Petersen *et al.*, industrialized countries spend 5%–10% of their public health expenditures on periodontal disease, dental caries and related dental care [11]. Unquestionably, one of the most common global diseases is dental caries [12].

A more pathogenic *Streptococcus* specie, *S. pyogenes* can be carried asymptomatically by humans but can cause mild to severe diseases, such as pharyngitis, tonsillitis, scarlet fever, cellulitis, erysipelas, rheumatic fever, post-streptococcal glomerulonephritis, necrotizing fasciitis, *etc*. (Table 1) [13]. It has been estimated that severe *S. pyogenes* infections lead to 517,000 deaths per year globally in addition to 233,000 deaths caused by rheumatic fever disease [14]. In United States alone 1800 invasive *S. pyogenes* disease-related deaths (necrotizing fasciitis and streptococcal toxic shock syndrome) are reported annually [15,16].

Another specie that most frequently has been linked to neonatal infections (early-onset and late-onset) such as sepsis, pneumonia and meningitis is *S. agalactiae* [17,18]. Late-onset neonatal infections (occurring at the age of 1–3 months) put infants at higher risk (as high as 20% even with proper antibiotic treatment) than early-onset neonatal infections of neonates (occurring within the first 24–48 h up to 7 days) [17]. In adults, *S. agalactiae* could cause peripartum choriomamniotitis, bacteremia, pneumonia, endocarditis, osteomyelitis, urinary tract infections, skin and soft tissue infections with immunocompromised individuals at highest risk (Table 1) [18,19].

Other important human pathogenic streptococci, *S. pneumoniae*, claimed the lives of 826,000 children under the age of five in year 2000 [20,21]. Globally, about 14.5 million episodes of invasive pneumococcal disease occur every year however mortality varies at 5%–35% depending on other factors (e.g., comorbidity, age, site of infection) [22]. In USA, annually 4 million episodes of pneumococcal diseases account for 445,000 hospitalizations and 22,000 deaths and *S. pneumoniae* is still the leading cause of bacteremia, meningitis, and pneumonia among all age groups (Table 1) [23].

Streptococci have a variety of potent virulence factors enabling them to cause such diverse infections [5]. Adhesins are one such factor because they play an important role in colonization [5]. Adhesins and virulence factors of streptococci have been reviewed extensively [5,6,24,25]. Carcinogenicity capacity of *S. mutans* is largely dependent on the ability of the bacteria to adhere and produce acid [12]. *S. mutans* glucosyltransferases assist in the adhesion process by synthesizing insoluble glucan from sucrose [12]. On the other hand, *S. pyogenes* produces extracellular proteins that have been shown to give rise to the remarkable virulence of the organism, triggering a nonspecific host immunological response [26]. Specific virulence factors assist *S. pyogenes* to attach to the host tissue, escape phagocytosis, and spread by infiltrating the host epithelial layers followed by colonizing [5,17,27,28,29]. In the case of *S. agalactiae*, major virulence and pathogenic factors enable the bacterium to stimulate sepsis syndrome, adhere to epithelial surface succeeding invasion, and avoidance of phagocytosis [30]. *S. agalactiae* attaches to host cells via fibronectin, fibrinogen and laminin [30]. For *S. pneumoniae*, a number of proteins, including hyaluronate lyase, pneumolysin, neuraminidases, the major autolysin, choline binding protein A, pneumococcal surface antigen A have been suggested to be virulence associated factors of this bacterium [31]. In addition, polysaccharide capsule is considered to be a key virulence factor [31].

**Table 1:** Demonstrated virulence factors of streptococci species, disease caused and the associated social and financial cost with the disease.

Organism	Diseases	Adherence Site	Estimated Cases/Costs
*S. mutans*	Dental caries	Tooth surface, other bacteria present in the biofilm on the surface of the tooth [5]	500 million visits to dentists and an estimated $108 billion spent on dental services in united states in 2010 [27]
	Dental plaque		
	Endocarditis		
*S. pyogenes*	Pharyngitis	Mucosal surfaces of pharynx, skin [25]	1–2.6 million cases of strep throat, erythromycin-resistant, invasive *S. pyogenes* causes 1300 illnesses and 160 deaths in united states each year. The total cost (medical and non-medical ) of group A streptococcal pharyngitis among school aged children in united states ranges from $224 to $539 million per year [27]
	Cellulitis		
	Streptococcal toxic-shock syndrome		
	Necrotizing fasciitis		
	Rheumatic fever		
	Sequela		
	Erysipelas glomerulonephritis		
*S. agalactiae*	Neonatal sepsis	Mucosal surfaces of vaginas and recta of pregnant women, skin [32]	Clindamycin-resistant *S. agalactiae* causes an estimated 7600 illnesses and 440 deaths yearly in U.S. 27,000 cases of severe *S. agalactiae* disease, such as blood infections or meningitis, occurred in 2011, causing 1575 deaths in U.S. [27]
	Meningitis		
	Systemic infection in immuno-compromised individuals		
*S. pneumoniae*	Otitis media	Mucous membranes of the nasopharynx [33]	Cases of resistant pneumococcal pneumonia result in about 32,000 additional doctor visits and about 19,000 additional hospitalizations and costs associated are approximately $96 million in U.S. [27]
	Bacteraemia		
	Pneumonia		
	Meningitis		
	Bronchitis		
	Sinusitis		
	Laryngitis		
	Epiglottitis		

### 1.3. Mechanism of Pathogenicity of Streptococcal Diseases

#### 1.3.1. Adhesion, Plaque, and Biofilm Formation of Streptococcal Species

To cause disease, a bacterial pathogen needs to meet several basic requirements. First, it must be able to adhere to the tissue surface and compete with the normal microbiota present on that surface [5,34,35]. Subsequently, for sustainable attachment, biofilms are developed and this may lead to invasion of the host tissue [6]. To establish biofilm, planktonic bacteria attaches to either inert or coated surfaces and this can be mediated by electrostatic contacts or bacterial surface adhesins [36]. Attachment is followed by proliferation of the primary colonizers and their co-aggregation with other planktonic bacteria, production of exopolysaccharide which stabilizes the architecture, leading to the maturation of the biofilm [36]. Sessile bacteria then could detach and form biofilms at different site [36,37,38]. Biofilm formation is not an attribute only specific to a few species, but a general ability of all microorganisms. Biofilm formation pathways are species specific, diverse, and dependent on environmental factors [39]. Although diverse, there are common features among all biofilms: (i) cells in the biofilm are glued together by an extracellular matrix made of exopolysaccharides, proteins, and occasionally nucleic acids; (ii) biofilm formation is initiated by environmental and bacterial signals; and (iii) biofilm offers bacteria protection from antibiotics and environmental stresses including immunological responses of the host [39]. Bacterial biofilms can build up on abiotic (plastic, glass, metal, *etc.*) or biotic (plants, animals, and humans) surfaces [34,38,40]. Mammalian-tissue colonizing species of *Streptococcus* live within biofilm in the natural environment [6,41,42].

Bacteria increase the expression of their outer cell surface adhesins when environmental conditions allow promoting cell-cell and cell-surface interaction [6,43]. Streptococci owe their success in colonization to their wide range of proteins expressed on their surfaces [5,6]. Surface adhesins facilitate interrelation with salivary, serum, extracellular matrix elements, host cells and other microbes [5,6]. Many of these adhesins are anchored to the cell wall peptidoglycan via their C-terminus or to the cell membrane via their N-terminal lipid (lipoproteins), and other adhesins remain surface localized through non-covalent interactions with other proteins or polysaccharides on the cell surface [6,44].

Most bacterial pathogens, including streptococci, have long filamentous structures known as pilli or fimbriae that are also involved in adhesion and biofilm formation [34]. In Gram-positive bacteria, hydrophobic components can be found: (i) covalently bound to cell wall, such as streptococcal M and F proteins, (ii) in the cytoplasmic membrane (e.g., lipoteichoic acid (LTA) of *S. pyogenes* or sialic acid of *S. agalactiae*) or (iii) located on the surface, like pilli or fimbriae [6,44,45]. Aside from adherence, biofilms are of significant importance as approximately 65% of human bacterial infections involve biofilms [45] including *Streptococcus* species (e.g., *S. mutans*, *S. pyogenes*, *S. agalactiae*, and *S. pneumoniae*) [34,40,41,46]. Clinically, biofilms are important because they reduce susceptibility of the bacteria to antimicrobials, prospering resistant bacteria leading to persistent infections [47,48].

The primary cause of dental caries is dental plaque which is a complex biofilm [41]. Broad spectrum of saliva proteins contribute to and initiate adhesion and dental biofilm formation [41,49,50]. Adhesion of *S. pyogenes* to various host cells is facilitated by the capsule and several factors embedded in the cell wall including M protein, LTA, and F protein [6,25,51]. M protein not only helps bacteria to attach to the host tissue but also inhibits opsonization by binding to host complement regulators and to fibrinogen [52]. A recent study has demonstrated that *S. pyogenes* pilus promotes pharyngeal cell adhesion and biofilm formation [53]. Altering surface hydrophobicity by sub-minimum inhibitory concentration of penicillin and rifampin reduces the adhesion of *S. pyogenes* to epithelial cells suggesting that surface-associated LTA will determine the surface hydrophobicity content of *S. pyogenes*, which consequently affects the bacteria’s interaction with mammalian host cells [54,55,56].

*S. agalactiae* produces several virulence factors such as adhesins [6]. These surface proteins and LTA of *S. agalactiae* bacterial cell wall contribute to the adhesion process mediating the invasion of eukaryotic cells [30]. Non-encapsulated *S. agalactiae* strains show increased adherence to eukaryotic cells [30]. *In vitro* studies have shown that *S. agalactiae* adheres to vaginal, buccal, endothelial and pulmonary epithelial cells [30]. Many clinical isolates of *S. mutans*, *S. pyogenes*, and *S. agalactiae* have been reported to be hydrophobic while their avirulent counterpart strains lacked this feature [57,58,59,60]. Studies have shown that *S. pneumoniae* adheres to abiotic surfaces, e.g., polystyrene or glass, and forms three-dimensional biofilm structures that are about 25 micrometers deep [34]. This three-dimensional structure enables the bacteria to survive for long periods within the bacterial community [34].

#### 1.3.2. Proton-Extrusion and Glycolysis of Streptococcal Species

Vital to the survival of bacteria is the regulation of the cytoplasmic pH as cellular activity requires a specific pH range [61]. Cytoplasmic pH is modulated by environmental pH, production, or consumption of internal protons, and transferring acids and bases across the plasma membrane [62]. The function of F-adenosine triphosphatase (F-ATPase) in streptococci is to regulate internal pH by pumping protons out of the cell [62,63]. The physiological role of streptococcal F_0_F_1_-ATPase is to alkalinize the cytoplasmic pH in the acidic pH range and to establish a proton reserve for a variety of secondary transport systems [64,65,66]. Streptococci are deficient in respiratory chains and are unable to produce a large proton gradient across the membrane, however, they make up for this lack by utilizing a range of basic transport systems [66]. For example, synthesizing a cytochrome-like respiratory chain, formation of adenosine triphosphate (ATP) from adenosine diphosphate (ADP) and inorganic phosphate by coupling the nicotinamide adenine dinucleotide hydrogen (NADH) oxidation with phosphorylation reaction [66,67,68]. Generally, ATPase in streptococci does not function as ATP synthase because of lack of a functional electron transport system; thus, it functions as hydrolase for proton movements coupled to ATP hydrolysis that are used for the generation of the proton gradient [66]. Streptococci utilize the glycolytic pathway to metabolize glucose to lactic acid [4,66]. Glucose is taken up, phosphorylated to glucose-6-phosphate through the phosphoenolpyruvate-dependent phosphotransferase system, and then converted to pyruvate, and eventually to lactic acid [66,69].

*S. pneumoniae* and oral streptococci could adapt to different environments and this capability is facilitated by ATPase regulating the intracellular concentration of solutes, including protons, and maintaining the pH homeostasis by proton extrusion [66,70]. Adherence is dependent: (i) on the synthesis of extracellular polysaccharides (mostly glucans) from the disaccharide sucrose through glucosyltransferases (GTFs) for *S. mutans*, and (ii) bacteria’s ability to produce acid by glycolysis and its tolerance to the produced acid [71]. *S. mutans* has the properties of acid production from sugar metabolism causing a drop in pH in dental plaque [72]. Low pH values in the plaque matrix leads to demineralization of tooth enamel, selection of acid-tolerant streptococci and eventually dental caries [72]. The glucans synthesized by GTFs promote the binding and accumulation of *S. mutans* and other bacteria on the tooth surface and contribute to the formation of biofilms [72,73,74,75]. *S. mutans* increases the proton-translocation, and F-ATPase activity when the environment’s pH drops, thereby this bacterium could withstand acidification influences [66,76,77]. F-ATPase transfers protons out of cells with the assistance of ATP hydrolysis to maintain its intracellular pH (e.g., more alkaline than the extracellular environment) [76]. F-ATPase enzyme is composed of two domains; (i) F_1_, the cytoplasmic catalytic domain; and (ii) F_0_, the proton-conducting membrane domain [67,78]. *S. mutans* does not produce catalase or cytochromes (thus a heme-based electron transport system) and so does not have oxidative phosphorylation linked to trans-membrane electron transport [66,79].

#### 1.3.3. Glucan Synthesis, Aggregation and Quorum Sensing of Streptococcal Species

Glucans interact with surface-associated glucan binding proteins of *S. mutans* to initiate colonization, cell-cell aggregation and the firm adherence of its cells to tooth surfaces [72,80]. *S. mutans* produces three types of GTFs: GTFB, GTFC, GTFD, and each of these enzymes are composed of two functional domains: (i) an amino-terminal catalytic domain (CAT); and (ii) a carboxyl-terminal glucan-binding domain (GBD) [81]. GTFB and GTFC, located on the cell surface, are encoded by *gtfB* and *gtfC* genes and GTFD is encoded by the *gtfD* gene [82]. Therefore, one of the strategies to control biofilm formation and dental caries is to inhibit the activity of GTFs: (i) GTFB (which synthesizes a polymer of mostly insoluble α1, 3-linked glucan); (ii) GTFC (which synthesizes a mixture of insoluble α-1,3-linked glucan and soluble α-1,6-linked glucan); and/or (iii) GTFD (which synthesizes water-soluble glucans rich in α-1,6-glucosidic linkages) [83,84].

Many streptococci use quorum-sensing systems to regulate several physiological properties, including the ability to incorporate foreign deoxyribonucleic acid (DNA), tolerate acid, form biofilm, and become virulent [85,86,87,88]. Quorum sensing, a strategy of cell-to-cell communication in a biofilm community, regulates unnecessary over-population and nutrient competition [89,90]. Bacterial activities including virulence gene expression within biofilms is regulated by the occurrence of quorum sensing [91]. This topic as well has comprehensively been discussed in review articles [87,92,93].

### 1.4. Treatment of Streptococcal Infection

Penicillin or one of its derivatives (e.g., amoxicillin and ampicillin) are the recommended antibiotic treatment for non-allergic patients diagnosed with *S. pyogenes* and *S. agalactiae* infections [27]. For allergic individuals, azithromycin and clarithromycin are recommended and in fact, azithromycin is prescribed more commonly than penicillin [94]. For severe *S. pyogenes* infections like necrotizing fasciitis and toxic shock syndrome, a combination of penicillin and clindamycin are prescribed [95]. *S. pyogenes* and *S. agalactiae* are not resistant to penicillin, but over time they have become resistant to clindamycin, tetracycline, vancomycin and macrolides (e.g., erythromycin, azithromycin and clarithromycin) [27]. Clarithromycin, clindamycin and vancomycin resistance among *S. pyogenes* and *S. agalactiae* strains are most concerning [27].

### 1.5. Antibiotic Resistance and Emerging Threats

Antimicrobial resistance is compromising the treatment of invasive infections including severe streptococcal infections [27]. This threat becomes significant in vulnerable patients (e.g., individuals undergoing chemotherapy, dialysis and organ transplants) due to infection-related complications [27]. This puts healthcare providers in the position to use antibiotics that may be more toxic to the patient, and frequently more expensive, leading to an increased risk of long-term disability and lower survival rates [27].

According to Frieden, director of the U.S. Center for Disease Control and Prevention (CDC), antimicrobial resistance is a serious health threat in the 21st century [27]. Infections caused by resistant bacteria are now on the rise and their resistance to multiple types and classes of antibiotics is worrisome [96]. The decrease in the rate of pathogen susceptibility to antibiotics has made it much more difficult to combat the infectious diseases [27]. The CDC’s 2013 report has prioritized drug-resistant *S. pneumoniae* as a serious threat, and erythromycin-resistant *S. pyogenes* and clindamycin-resistant *S. agalactiae* as concerning threats [27].

### 1.6. Possible Alternatives for Classical Antibiotics

Plants produce diverse secondary metabolites or phytochemicals, most of which are isoprenoids and polyphenols and their oxygen-substituted derivatives such as tannins that could be raw materials for future drugs [97]. Herbs and spices contain useful medicinal compounds including antibacterial chemicals, and researchers have found that many of these compounds inhibit the growth of pathogenic bacteria [97]. Accordingly, experimental observations have shown that herbal preparations are active against many of the pathogens (Table 2).

From the period of 1981 to 2006, 109 new antibacterial drugs were approved for treatment of infectious diseases of which 69% originated from natural products, and 21% of antifungal drugs were natural derivatives or compounds mimicking natural products [98]. Various medicinal plants have recently been tested for their antimicrobial activity and all have proven that phytochemicals, particularly polyphenols, exhibit significant antibacterial activity against *Streptococcus* species (Table 3).

## 2. Anti-Streptococcal Attributes of Phytochemicals

Many fruits and plants have shown to possess anti-streptococcal effects (Table 3). Folklore medicinal plants have long been used for the treatment of *S. pyogenes* infections (Table 2) including pharyngitis. For example cashew plant (*Anacardium occidentale*), stickwort (*Agrimonia eupatoria*), mountain daisy (*Arnica montana*), bayberry (*Myrica cerifera*), soft leafed honeysuckle (*Lonicera japonica*), cuajilote (*Parmentiera aculeate*) or baobab (*Adansonia digitata*) [99,100,101,102,103,104], (Table 2). Particularly more attention has been given to anti-streptococcal effects of phytochemicals against *S. mutans* due to its cariogenic properties. A wide range of commercial and freshly prepared polyphenolic rich extracts (70% propanone) of various teas including green and black tea, lemon, cinnamon, hibiscus, peppermint, grape seed, sloe berry skin, cocoa, blackberry, pomegranate skin, blackcurrant, hawthorn berry skin, red and white wine was tested for their anti-streptococcal activity against oral streptococci (various strains of *S. mutans*, *S. oralis*, *S. gordonii*, *S. salivarius*, *S. sanguis*) [105]. All the tested products exhibited their minimum inhibitory effect at concentrations ranging 0.25–32 mg/mL against *Streptococcus* species [105]. Red grape seed propanone extract was most potent against *S. mutans* and Agro tea extract least effective with minimum inhibitory concentration of 0.5 mg/mL and 32 mg/mL respectively [105]. Phytochemicals, although very limited, also have been shown to hinder the growth of *S. agalactiae* [106,107,108,109,110,111,112]. Aqueous, ethanolic and chloroform extracts of bael, Indian gooseberry, moringa, neem, Chinese mahogany exert their minimum inhibitory effects at concentrations ranging from 0.15 mg/mL to 10 mg/mL against *S. agalactiae*, chloroform extract of Chinese mahogany being the most active one [111]. In a study by Nguelefack *et al.* ethyl acetate bark extract of *Distemonanthus benthamianus* at Minimum Bactericidal Concentration (MBC) of 4096 µg/mL was effective against *S. agalactiae* and its phytochemical profile was indicative of presence of flavonoids and phenolics and absence of sterols, triterpenes and alkaloids [113]. Moderate inhibitory effect of wild *Asparagus racemosus* ethanol extract at concentration of 500 μg/disc was also reported for *S. agalactiae* [114].

### 2.1. Phytochemicals with Inhibitory Activities against Adhesion, Plaque, and Biofilm Formation

Phytochemical-rich extracts and their associated pure compounds have repeatedly shown inhibitory effects against adhesion, plaque, and biofilm formation of streptococcal species (Table 4 and Table 5). High molecular weight non-dialysable materials extracted from cranberry juice (NDM) exhibit adhesion reduction activity in a dose-dependent manner at concentrations of 66–1330 µg/mL against *S. sobrinus* [115]. In another study, the ethanolic extract of *Helichrysum italicum* at concentrations of 15–31 µg/mL inhibited the sucrose-dependent adherence of *S. mutans* cells to a glass surface by 90% to 93% [116]. Cranberry juice powder (25%) at 500 μg/mL concentration inhibited the biofilm formations of *S. sobrinus* and *S. sanguinis* significantly [117]. In the same study, cranberry juice powder decreased the cell surface hydrophobicity of *S. mutans* and *S. sobrinus* 6715 by more than 40% [117].

**Table 2:** Folklore medicine used for Streptococcal diseases or diseases with similar clinical Presentations.

Folklore Medicinal Plant	Targeted Disease Condition
*Agrimonia eupatoria* L.	Acute sore throat and chronic nasopharyngeal catarrh [118,119]
*Arnica montana* L.	Inflammation of oral, throat region [99,120,121]
*Lonicera* japonica Thunb.	Erysipelas, pharyngitis, upper respiratory infection [100]
*Morella cerifera* (L.) Small	Cold and sore throat [101,122]
*Parmentiera aculeate* (Kunth) Seem	Otitis media [123]
*Adansonia digitata* L.	Otitis media [102,124]
*Anacardium occidentale* L.	Sore throat [103,125]
*Uvaria chamae* P. Beauv.	Sore throat [64,126,127]
*Adansonia digitata* L.	Inflamed gums and infected teeth [128]
*Carica papaya* L.	Toothache [129]
*Hyoscyamus niger* L.	Toothache [130,131]
*Eucalypthus camaldulensis* Dehn.	
*Anacardium occidentale* L.	Toothache, sore gums [132]
*Annona reticulata*	Toothache [133,134]
*Annona squamosa* Linn	
*Uvaria chamae* P. Beauv	Inflamed gums [135]
*Abutilon indicum* (L.) Sweet, *Baliospermum axillare* Blume, *Blumea lacera* (Burm. f.) DC., *Canna indica* L., *Ocimum tenuiflorum* L., *Oroxylum indicum* (L.) Vent., *Polygonum aviculare* L., *Solanum indicum* Linn., *Vernonia patula* (Aiton) Merrill [136]	For the relief of symptoms of bronchitis, pneumonia, influenza [136]
*Vigna radiata* (L.) R. Wilczek *Andrographis paniculata* (Burm. f.) Wall. ex Nees [137,138]	Treatment of sepsis [137,138]

**Table 3:** Inhibitory effects of phytochemicals against selected *Streptococcus* species.

Species	Strain	Plant	EM	MIC, IZD	Ref.
*S. pyogenes*		*Passiflora foetida* L.	EE, ACE	100–400 µg/mL, 10–20 mm	[139]
	ATCC 19615	*Ageratum conyzoides* L.	AE, EE, ME	1–2 mg/mL	[140]
		*Laggera tomentosa* Sch-Bip			
		*Syzygeum guineense* DC.			
		*Cordia africana* Lam.			
		*Ferula communis* L.			
		*Discopodium peninervum* Hochst			
		*Olea europea* subsp. cuspidate			
		*Crescentia cujete* L.	CEE	5 mg/mL	[141]
	Cl	*Uvaria chamae* P. Beauv	CAE, HAE	9–12 mm, 100 µg/mL	[127]
		*Vernonia amygdalina* Del.			
		*Garcinia kola* Heckel			
	CI	*Uvaria chamae* P. Beauv	CDEE, HEE	6–21 mm, 100 µg/mL	[127]
		*Vernonia amygdalina* Del.			
		*Garcinia kola* Heckel			
		*Aframomum melegueta* Schum.			
	CI	*Zingiber officinale* Roscoe	EE	2–6 mm, 0.0005–0.389 µg/mL	[142]
	CI	*Garcinia kola* Heckel	EE	0.0005–0.44 µg/mL	[142]
	CI	*Coccinia grandis* (L.) Voigt	HE	5.5–7 mm	[143]
	CI	*Eucalyptus globulus* Labill.	ME	32–64 mg/L	[144]
	HITM 100	*Quercus ilex* L.	BE, EAE	10 mm, 512 μg/mL	[106]
	CI	*Prunus armeniaca* L.	CEE, BE	250 μg/mL	[145]
	ATCC 19615	*Capsicum chinense* Jacq.	AE	15–34 mm	[146]
	ATCC 19615	*Allium sativum* L.	AE	29 mm	[146]
	CI	*Spilanthes acmella* Murr.	CHE	256 μg/mL	[147]
	CI	*Cinnamomum zeylanicum* Garcin ex Blume	EO	6.25 µL/mL	[107]
	CI	*Thymus vulgaris* L. *Syzygium aromaticum* (L.) Merr. & L.M. Perr	EO	12.5 µL/mL	[107]
	CI	*Sechium edule* (Jacq.) Sw.	EE	10–15 mm	[108]
*S. mutans*	ATCC 25175	*Coffea canephora* Pierre ex Froehner	AE	5 mg/mL	[148,149]
	ATCC 25175	*Baeckea frutescens* L.	75% ME	14–22 mm, 20, 50 mg/mL	[150,151]
		*Glycyrrhiza glabra* L.			
		*Kaempferia pandurata Roxb.*			
		*Physalis angulata* L.			
		*Quercus infectoria* Oliv.			
	MTCC-890	Nut gall (*Quercus infectoria*)	Petro, ether Water, metahnol	12–23 mm	[150]
	UA159	*Rheedia brasiliensis* Planch. & Triana	HE	1.25–2.5 μg/mL	[152]
	UA159	*Camellia sinensis* (L.) Kuntze	Epigallocatechin gallate by HPLC	31.25 μg/mL	[153]
	ATCC 700610	*Prosopis spicigera* Linn.	ACE, CHE DEE, EAE, EE, ME, PEE	9.76–1250 μg/mL	[154]
	ATCC 700610	*Zingiber officinale* Roscoe	ACE, CHE DEE, EAE, EE, ME, PEE	625–2500 μg/mL	[154]
		*Trachyspermum ammi* (L.) Sprague ex Turrill	CE, PEE	40–320 μg/mL	[155]
	ATCC 25175	*Siraitia grosvenorii* (Swingle) A. M. Lu & Zhi Y. Zhang	commercial extract	6 μg/mL	[156,157]
*S. puenomonia*	CI	*Zingiber officinale* Roscoe	EE	0.001–0.7 µg/mL	[142]
	serotype 6B	*Agaricus blazei* Murill	AE		[158]
	serotype 6B	*Plantago major* L.	AE	0.48 mg/kg	[159]
	ATCC 49619, penicillin resistant and sensitive clinical strains	*Garcinia afzelii* Engl.	90% EE	6– >1500 µg/mL	[160]
		*Andira inermis* (W. Wright) Kunth ex DC.			
		*Keetia hispida* (Benth.) Bridson			
		*Uapaca togoensis* Pax *Combretum molle* (R. Br. x. G. Don)			
		*Erythrina senegalensis* DC.			
		*Piliostigma thonningii* (Schum.)			
	CI	*Garcinia kola* Heckel	EE	0.00008–1.7 µg/mL	[142]
	CI	*Eucalyptus globulus* Labill.	ME	16–32 mg/L	[144]
	ATCC 49619	*Salvia tom entosa* Mill.	EO	2.25 mg/mL	[161]
	CI	*Thymus vulgaris* L. *Cinnamomum zeylanicum* Garcin ex Blume	EO	6.25 µL/mL	[107]
	Antibiotic resistant strains	*Eucalyptus globulus* Labill.	CAE	0.7 mg/mL	[162]
	CI	*Syzygium aromaticum* (L.) Merr. & L.M. Perr	EO	12.5 µL/mL	[107]
	ATCC 49619	*Euphorbia hirta* L.	AE, EE, ME	6–11 mm, 60–80 mg/mL	[163]
		*Laggera tomentosa* Sch-Bip	ME, AE	1–2 mg/mL	[140]
		*Syzygeum guineense* (Wild.) DC.			
		*Cordia africana* Lam.			
		*Ferula communis* L.			
		*Olea europea* subsp. *cuspidate*			
*S. agalactiae*	NCIM 2401	*Ficus tsiela* Roxb.	EE	9.5 mm	[109]
	NCIM 2401	*Hibiscus sabdariffa* L.	AE	9 mm	[109]
	HITM 80	*Quercus ilex* L.	BE, EAE	8–11 mm, 512 μg/mL	[106]
	CI	*Syzygium aromaticum* (L.) Merr. & L.M. Perr *Cinnamomum zeylanicum* Garcin ex Blume	EO	12.5 µL/mL	[107]
	CI	*Thymus vulgaris* L.	EO	6.25 µL/mL	[107]
	CI	*Spathodea campanulata* P. Beauv.	CAE, CME	2–7 mm	[110]
		*Tridax Procumbens* L.			
		*Sechium edule* (Jacq.) Sw.	EE	15 mm	[108]

Abbreviations: ACE; Acetone Extract, AE; Aqueous Extract, BE; Butanolic Extract, CAE; Crude Aqueous Extract, CDEE; Cold Ethanolic Extract, CE; Crude Extract, CHE; Chloroform Extract, CEE; Crude Ethanolic Extract, CI; Clinical Isolate, CME; Crude Methanolic Extract, DEE; diethyl ether extract, EAE; Ethyl Acetate Extract, EE; Ethanolic Extract, EM; Extraction Method, EO; Essential Oil, HAE; Hot Aqueous Extract, HE; Hexane Extract, HPLC; High Performance Liquid Chromatography, IZD; Inhibition Zone Diameter, ME; Methanolic Extract, MIC; Minimum Inhibitory Concentration, PE; Petroleum Extract, PEE; Petroleum Ether Extract, Ref.; References.

**Table 4:** Inhibitory effects of phytochemicals against adhesion, biofilm formation and hydrophobicity.

Plant/Fruit Name	Bioactive Compounds and EM	Bacterial Strain	Concentration and Assay Type	Results	Ref.
Maidenhair tree (*Ginkgo biloba* L.) South African geranium (*Pelargonium sidoides* DC.) Cranberry (*Vaccinium macrocarpon* Aiton)	Purified PAC, AE, AEE, ME	*S. pyogenes* DSM 2071	*P. sidoides* 40% *G. biloba* 100% Adhesion reduction at 3 h incubation time	*P. sidoides* 40% *G. biloba* 25%	[164]
Cranberry (*Vaccinium macrocarpon* Aiton)	High MW non-dialyzable materials Juice powder 25% concentration, dissolved in water	*S. mutans* MT 8148R, JC2, Ingbritt, ATCC 10449 *S. criceti* E49 *S. oralis* ATCC 10557 *S. mitis* ATCC 9811 *S. gordonii* Challis	100–500 μg/mL Inhibition of biofilm formation	Significant inhibition	[117]
		*S. mutans* MT 8148R, JC2, Ingbritt *S. sobrinus* 6715	Effect on hydrophobicity	40%–60% reduction	
Cocoa (*Theobroma cacao* L.)	PP fractions Oligomers: Monomer MW 290 Dimer MW 578 Tetramer MW 1154 Pentamer MW 1442 HE	*S. mutans* NCTC 10449 CI of *S. sanguinis* LDI1	35 μM Biofilm biomass reduction after 4 h	In absence of sucrose *S. sanguinis* 48% *S. mutans* 68%	[165]
				In presence of sucrose *S. sanguinis* 79% *S. mutans* 44%	
Cranberry (*Vaccinium macrocarpon* Aiton)	High MW non-dialysable material, CJ	*S. sobrinus* 6715	1.33 mg/mL Adhesion to glucan or fructan coated hydroxyapatite reduction	95%	[115]
Red grape (*Vitis vinifera* L.) Pine bark	Red grape marc extract (GME): 20% PP, 3% A, Red wine extract (RWE): 95% PP Pine bark extract (PBE) Commercial preparation	*S. mutans* ATCC 25175	2 mg/mL Adhesion to glass surface Inhibition	GME significant inhibition, RWE, PBE effective at >4 mg/mL	[166]
Blueberry (*Vaccinium myrtillus* L.) Small cranberry (*Vaccinium oxycoccos* L.) Lingonberry (*Vaccinium vitis-idaea* L.) Cloudberry (*Rubus chamaemorus* L.) Crowberry (*Empetrum nigrum* L.) Blackcurrant (*Ribes nigrum* L.) Sour cherry (*Prunus cerasus* L.)	Molecular size of fractions; F1 <10 kDa, F2 10–100 kDa, F3 >100 kDa F2 and F3: polyphenol macromolecular complexes: PACs, polyhydroxy flavonoids AE, CJ	CI of *S. pneumoniae* SB 53845 *S. agalactiae* B133 III R	Binding activity of bacterial cells	*S. pneumonia* bound to fraction FI of cranberry and bilberry juices *S. agalactiae* bound to bilberry juice and cranberry fractions FII and FIII and to all fractions of cranberry juice and lingonberry	[112]
Clove (*Syzygium aromaticum* (L.) Merr. & L.M. Perr)	CAE	*S. mutans* ATCC 25175	20 mg/mL Precent cell-surface hydrophobicity	0.3% ± 0.1%	[167]
	CAE	*S. mutans* ATCC 25175	20 mg/mL Adherence inhibition	100%	
	CME	*S. mutans* ATCC 25175	20 mg/mL Percent cell-surface hydrophobicity reduction	25.2% ± 4.7%	
	CME	*S. mutans* ATCC 25175	15 mg/mL Adherence inhibition	100%	
Cocoa (*Theobroma cacao* L.)	Bean husk extract 12.6% PP compounds 30% EE	*S. mutans* MT8148	1 mg/mL Adherence to saliva-coated hydroxyapatite inhibition	31%	[168]
		*S. mutans* MT8148	1 mg/mL Plaque formation inhibition	Significantly inhibited	
Guava (*Psidium guajava* L.)	Quercetin-3-*O*-alpha-l-arabinopyranoside (guaijaverin) ME	*S. mutans* MTCC1943	2 mg/mL Percent cell hydrophobicity	20%	[169]
Cranberry (*Vaccinium macrocarpon* Aiton)	PP fraction	*S. sobrinus* 6715 *S. sobrinus* B13 *S. mutans* MT8148R *S. mutans* JC2	500 μg/mL Hydrophobicity reduction	*S. sobrinus* 6715 90% *S. sobrinus* B13 85% *S. mutans* MT 8148R 90% *S. mutans* JC2 65%	[170]
Devil’s horsewhip (*Achyranthes aspera* L.)	AE, BE, ME, PEE	CI of *S. mutans*	125 µg/mL Biofilm inhibition	Complete to partial biofilm inhibition	[171]
Meswak (*Salvadora persica* L.)	ACE, AE, CHE, EE, ME	CI of *S. mutans*	2.6 mg/mL Biofilm inhibition	significant inhibition	[172]
Indian gooseberry (*Emblica Officinalis* L.)	CE, EF	*S. mutans* MTCC 497	39.04 µg/mL CE, 78.08 µg/mL ethanolic fraction Biofilm inhibition	50% inhibition	[173]
			156 µg/mL CE and 312.5 µg/mL ethanolic fraction Adherence inhibition	50% inhibition	
			Hydrophobicity reduction	Partial reduction	
Papaya (*Carica papaya* L.)	Fermented papaya preparation (FPP) Alkaloids Flavonoids Glucosides Anthraquinones	*S. mutans* 25175 *S. mitis* 6249	50 mg/mL Percent hydrophobicity	*S. mutans*: 1.01% *S. mitis*: 7.66%	[174]
Curry (*Helichrysum Italicum* G. Don)	Apigenin Luteolin Gnaphaliin Naringenin Pinocembrin Tiliroside EE	*S. mutans* ATCC 35668 *S. salivarius* ATCC 13419 *S. sanguis* ATCC 10556	16–31 μg/mL Adherence to glass surface inhibition	90%–93%	[116]
			sub-MIC 8–31 μg/mL Cell-surface hydrophobicity reduction	90%	

Abbreviations: A; Anthocyanin, ACE; Acetone Extract, AE; Aqueous Extract, AEE; Aqueous Ethanolic Extract, BE; Butanolic Extract, CAE; Crude Aqueous Extract, CE; Crude Extract, CHE; Chloroform Extract, CI; Clinical Isolate, CJ; Concentrated Juice, CME; Crude Methanolic Extract, EE; Ethanolic Extract, EF; Ethanolic Fractions, EM; Extraction Method, FPP; Fermented Papaya Preparation, GME; Red Grape Marc Extract, HE; Hexane Extract, kDA; Kilodalton, ME; Methanolic Extract, MW; Molecular Weight, PAC; Proanthocyanidin, PBE; Pine Bark Extract, PEE; Petroleum Ether Extract, PP; Polyphenol, Ref.; References, RWE; Red Wine Extract.

**Table 5:** Inhibitory effects of pure phytochemicals against adhesion, biofilm formation, quorum sensing and hydrophobicity.

Bioactive Compounds	Bacterial Strain	Concentration and Assay Type	Results	Ref.
(−)-Epicatechin (−)-epicatechin-3-*O*-gallate (−)-epigallocatechin (−)-epigallocatechin-3-*O*-gallate	*S. pyogenes* DSM 2071	30 μg/mL Adhesion reduction to HEp-2 cells	(−)-epigallocatechin 15% (−)-epigallocatechin-3-*O*-gallate 40%	[164]
Morin	*S. pyogenes* MGAS 6180	225 μM Biofilm biomass reduction	50%–60%	[175]
Ursolic acid (UA) Oleanolic acid (OA)	*S. mutans* UA159 *Actinomyces viscosus* ATCC 15987	1024 μg/mL Adherence inhibition to tooth surface	Complete inhibition	[176]
EGCG	ComC-deficient *S. mutans*	0.25 mg/mL Biofilm inhibition	81% Biofilm inhibition	[177]
		QS inhibition	Partial inhibition	

Abbreviations: ComC; competence factor, EGCG; Epigallocatechingallate, HEp-2; Human Epithelial Type 2 (Hep-2) Cells, OA; Oleanolic Acid, QS; Quorum Sensing, Ref.; References, UA; Ursolic Acid.

In a different study, anti-adhesion, biofilm inhibition and eradication activity of the two-terpenoids, ursolic acid (UA) and oleanolic acid (OA), were examined. UA and OA showed a Minimum Inhibitory Concentration (MIC) of 256 µg/mL and 1024 µg/mL against *S. mutans* UA159, respectively [176]. The Minimum Bactericidal Concentration (MBC) for UA and OA against the same bacterium were 256 µg/mL and >1024 µg/mL correspondingly [176]. Microtiter plate biofilm assay showed that sub-MIC dose of the compounds inhibited the biofilm formation [176]. Gallic acid at 1–4 mg/mL concentration inhibited up to 70% of *S. mutans* biofilm establishment [178]. Gallic acid, quercetin, and tannic acid all produced significant biofilm inhibition attributes against *S. mutans* however gallic acid was most potent [179]. Methyl gallate at concentrations of 1–4 mg/mL rendered biofilm formation of *S. mutans* to up to 80% [178]. Green and oolong tea contain substantial quantities of gallic acid and epigallocatechin gallate and have exhibited slight inhibition effect on the attachment of *S. mutans* and other oral bacterial to collagen, tooth surfaces and gingival cell line [180]. In the same study, fermented tea with high tannin content opposed to green tea and oolong tea had shown more activity towards attachment of *S. mutans* and other oral bacterial to collagen, tooth surfaces and gingival cell line [180].

Adhesion of *S. mutans* to the tooth surface was hindered after treatment with UA at 256 µg/mL [176]. Sub-MIC dose of UA also affected the adhesion consequently hindering the biofilm formation [176]. UA moreover eradicated the biofilm cells at concentrations of 500–2000 µg/mL [176]. Polyphenolics-rich tea extract at concentrations as low as 1–4 mg/mL prevented the attachment of *S. mutans* to collagen coated hydroxyapatite beads [181].

In another study, the effect of cocoa polyphenol fractions on *S. mutans* biofilm reduction in the absence and presence of sucrose were measured. At 35 μM concentration and after 4 h, biofilm mass was reduced to 68% in the absence of sucrose and to 44% in the presence of sucrose [165]. Biofilm of *S. mutans* on saliva coated hydroxyapatite surface was preformed and then treated (60 s) with purified proanthocyanidin (PAC)-containing fraction of cranberry (various degree of polymerization) [182]. At concentrations of 100 µM (single or combined fractions in 1:1 ratio), confocal 3D images show distorted architecture and deficient biofilm accumulation suggestive of reduced biomass and thickness of adherent bacteria and EPS [182]. Expressions of 119 genes of *S. mutans* within biofilm were altered post exposure to PAC-rich fractions of cranberry [182]. The expression of genes particularly related to adhesion, acid stress tolerance, glycolysis and other cellular activities during biofilm development were downregulated [182]. Structure activity relationship analysis revealed that PAC oligomers with more than eight epicatechin units exhibit higher anti-adhesion effects up to 85% against *S. mutans* however the increase in potency is not proportional [182]. This not only is associated with degree of polymerization but may also be associated with number and location of A-type linkages in the oligomers, and type of interflavan bonds [182].

The anti-adhesive properties of root extract of *Pelargonium sidoides* have been studied against *S. pyogenes* attachment to human epithelial type 2 (HEp-2) cells [164]. Results have shown that after pre-treatment of *S. pyogenes* with methanol insoluble and methanol soluble fractions of the extracts of *Pelargonium sidoides* at concentrations of 30 µg/mL, adhesion of the pathogen to HEp-2 cells was inhibited up to 30% to 35% [183]. To characterize the anti-adhesive constituents of these fractions, comparative chemical studies were performed. The study revealed that the proanthocyanidins content of the fraction was of prodelphinidin nature, and inhibition of the adhesion was in a specific rather than non-specific manner [164,183]. Successful inhibition of adhesion and hydrophobic interactions could reduce and or prevent sore throat caused by *S. pyogenes* [164]. It has been suggested that polymeric flavonoids or other large molecule polyphenols may exhibit higher anti-adhesion effects against streptococci [180]. Coffee high molecular weight fraction nearly completely (91%) hindered the adhesion of *S. mutans* [184].

**Figure 1:**
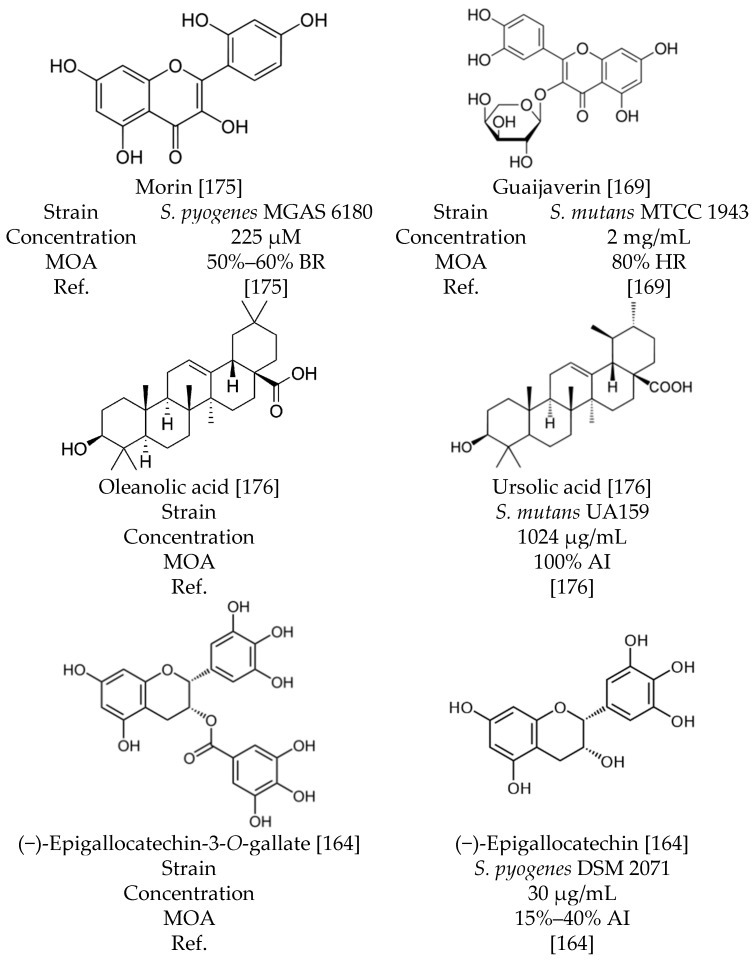
Chemical structure of polyphenols with inhibition activity against adherence, biofilm biomass and hydrophobicity. Abbreviations: AI; Adherence Inhibition, BR; Biofilm Biomass Reduction, HR; Hydrophobicity Reduction, MOA; Mode of Action, Ref.; References.

Similarly, a study on the binding activity of *S. pneumoniae* and *S. agalactiae* to different molecular size fractions (F1, F2, F3) of *Vaccinium* family polyphenols found that binding was highest to wild cranberry (*Vaccinium oxycoccos*) [112]. *S. pneumoniae* cells bound mostly to cranberry juice low-molecular size fraction (F1) and *S. agalactiae* cells to high-molecular size fraction (F3) [112]. *S. pneumoniae* bound to F1 of bilberry and cranberry juices and *S. agalactiae* attached most actively to F2 and F3 of berry and juice preparations belonging to *Vaccinium* species [112]. Phytochemical analysis has shown that F2 and F3 fractions contain polyphenol macromolecular complexes, including proanthocyanidins and polyhydroxy flavonoids [112]. At sub-MIC level of 2 mg/mL red grape marc extract, composed of 20% polyphenols and 3% anthocyanin, inhibited the adherence of *S. mutans* and *Fusobacterium nucleatum* cells to glass surface [166]. Morin, a flavonol, reduced biofilm biomass of *S. pyogenes* at concentrations exceeding 225 μM up to 65% [175]. Epigallocatechin gallate (EGCG) of *Camellia sinensis* has various physiological effects on *S. mutans* UA159 (Figure 1) and has been proven to inhibit the enzymatic activity of glucosyltransferases, F_1_F_0_-ATPase, lactate dehydrogenase, biofilm formation and growth [153].

### 2.2. Phytochemicals with Inhibitory Activities against F-ATPase and Glycolytic pH-drop

**Figure 2:**
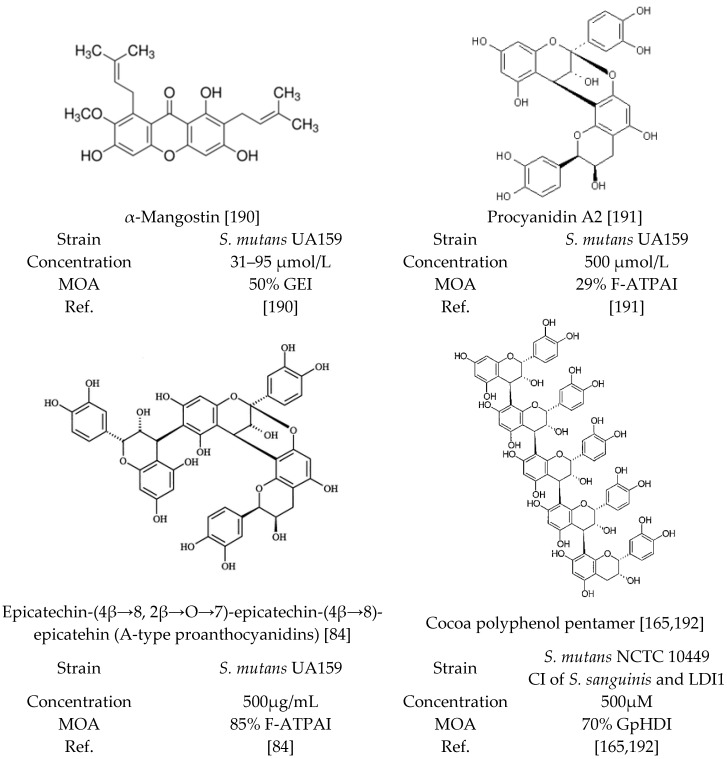
Chemical structure of polyphenols with inhibition activity against F-ATPase, glycolytic enzymes and glycolytic pH-drop. Abbreviations: CI; Clinical Isolate, F-ATPAI; F-ATPase Activity Inhibition, GEI; Glycolytic Enzymes Inhibition, GpHDI; Glycolytic pH-Drop Inhibition, MOA; Mode of Action, Ref.; References.

Phytochemical-rich extracts not only possess anti-adhesion, anti-plaque and anti-biofilm attributes, but also have demonstrated inhibitory effects on streptococcal species F-ATPase and glycolytic pH-drop activities (Table 6, Figure 2). Plants and fruits have been studied for their anti-streptococcal effects and fruits such as cranberry (*V. macrocarpon*), cocoa (*Theobroma cacao*), babchi (*Psoralea corylifolia*), mangosteen (*Garcinia mangostana*) and grape (*Vitis vinifera*) have shown inhibitory effects on F_0_-ATPase and F_1_-ATPase, glucosyltransferases (GTFB and GTFC) and acid production activities of *S. mutans* [80,84,165,185]. The lack of inhibitory activity of monophenolic compounds suggest that the inhibition of F_1_–F_0_-ATPase by phenolics require two or more phenolic structures [186]. The flavones have also been shown to interact with other ATPases, such as Ca^2+^-ATPase [187] and Na^+^/K^+^-ATPase [188], in addition to their inhibitory effects on F_1_–F_0_-ATPase [189]. Glycolysis of *S. mutans* is inhibited by α-mangostin leading to indirect inhibition of respiration by α-mangostin [190]. Glucan production by GTFs and F-ATPase is inhibited by α-mangostin suggesting that *S. mutants* can be eliminated selectively [190].

Analysis of low molecular weight cranberry polyphenols against glucosyltransferases, acid production and F-ATPase activity of *S. mutans* UA159 has suggested that compounds like phenolic acids have no inhibitory effect on these virulence factors [84]. Quercetin, quercetin-3-*O*-glucoside, quercetin-3-*O*-galactoside, quercetin-3-*O*-arabinofuranoside, quercetin-3-*O*-rhamnoside, myricetin, PAC-monomer, PAC-dimer, and procyanidin A2, at the concentrations of 500 µM inhibited the enzymatic activity of the proton-translocating F-ATPase to some degree [191]. Myricetin, procyanidin A2 and the combination of the two were most effective inhibitors with 32%, 29% and 43% inhibition against F-ATPase activity, respectively [192]. The flavonoids, particularly myricetin, procyanidin A2 and the combination of the two significantly interrupted the glycolytic pH-drop by *S. mutans* cells; however, epicatechin, myricetin-3-*O*-rhamnoside, caffeic acid, chlorogenic acid had no effect [191]. In presence of cocoa polyphenol pentamer, the terminal pH is increased to 4.67 ± 0.09 within 20 min while in untreated pH was as low as 4.50 ± 0.08 (*S. mutans* converts sucrose to acid and lowers the pH) [165]. These results suggest that 500 µM cocoa polyphenol pentamer reduced the rate of acid production, at pH 7.0, by 30% [165].

### 2.3. Phytochemicals with Inhibitory Activities against Glucosyltransferases, Aggregation, and Quorum Sensing

Moreover, phytochemicals-rich extracts have been reported for their inhibitory properties against glucosyltransferases, aggregation, and quorum sensing attributes of streptococcal species (Table 7). Low molecular weight polyphenols of cranberry reduced the glucan synthesis of *S. mutans* cells by GTFB and GTFC [191]. At 500 µM, the inhibition activities of the tested polyphenols varied from 15%–45% (epicatechin 15%, myricetin-3-*O*-rhamnoside 20%, procyanidin A2 30%, quercetin-3-*O*-arabinofuranoside 35% and quercetin-3-*O*-arabinofuranoside in combination with procyanidin A2 45%) [191]. It is notable that theaflavin of green tea at 10 mM inhibited the GTF activities of *S. mutans* significantly [193].

The effects of fractions (F1, F2, and F3) of juice concentrates of bilberry (*Vaccinium myrtillus*), lingonberry (*Vaccinium vitis-idaea*), cloudberry (*Rubus chamaemorus*), crowberry (*Empetrum nigrum* and *hermaphroditum*), apple (*Malus domestica*), and blackcurrant (*Ribes nigrum*) on anti-coaggregation and anti-aggregation activities of dental plaque bacteria have been tested [194]. Test has been done on the pairs of *S. mutans* IH 113728 with the two strains of *Actinomyces naeslundii* (AHP 28639 and AHP 28651) and *S. mutans* IH 113728 with the two strains of *Fusobacterium nucleatum* (AHN 23952 and AHN 23937) [194]. The anti-aggregation and anti-coaggregation activity was found in F2 and F3 of bilberry, blackcurrant, and crowberry and lingonberry juices [194]. Also, F2 and F3 of crowberry at 48 mg/g of Solid Solubles (SS) showed anti-co-aggregation against some of the pairs at 91% and 86%, respectively [194]. The anti-aggregation activity was detected in all bacterial pairs with fraction F2 of bilberry, crowberry and lingonberry juices [194]. The anti-aggregation was mainly achieved with a berry concentration of 48 mg/g of SS [194]. Analysis of composition of the juice fractions showed that F2 and F3 were composed of macromolecular polyphenol complexes, PAC, polyhydroxy flavonoids [194]. Absolute co-aggregation inhibition and anti-aggregation activity were achieved with the F2 of bilberry juice at the concentration of 48 mg/g of SS [194].

**Table 6:** Inhibitory effects of phytochemicals on F-ATPase activity and glycolytic pH-drop.

Plant	Bioactive Compounds and EM	Bacterial Strain	Concentration and Assay Type	Results	Ref.
Cranberry (*Vaccinium macrocarpon* Aiton)	FLAV A PAC	*S. mutans* UA159	PAC 500 µg/mL FLAV 125 µg/mL A 200 µg/mL F-ATPase activity inhibition	PAC alone or in combinations >85% FLAV 20%	[84]
			500 µg/mL Glycolytic pH-drop	PAC alone or in combinations pH 4.7–4.9	
Cranberry (*Vaccinium macrocarpon* Aiton)	Low MW PP	*S. mutans* UA159	500 µg/mL F-ATPase activity inhibition	Myricetin 32% procyanidin A2 29% Myricetin + procyanidin A2 43%	[191]
			Glycolytic pH-drop	Significant disruption	
Cocoa (*Theobroma cacao* L.)	Oligomers: Monomer MW 290 Dimer MW 578 Tetramer MW 1154 Pentamer MW 1442 HE of PP fractions	*S. mutans* NCTC 10449 *S. sanguinis* LDI 1, CI	500 µM pentamer Glycolytic pH-drop	30%	[165]
Red wine grape (*Vitis vinifera* L.)	Gallic acid Catechin Epicatechin Procyanidin B1 Procyanidin B2 Resveratrol Fermented	*S. mutans* UA159	125 µg/mL F-ATPase activity inhibition	30%–65%	[195]
			500 μg/mL Glycolytic pH-drop	Significant inhibition	
Green tea *Camellia sinensis* (L.) Kuntze	EGCG EE	*S. mutans* UA159	15.6 μg/mL Glycolytic pH-drop	Significant inhibition	[153]
Methuselah’s beard (*Usnea longissima* Ach.)	Herbo-metallic preparations	*S. mutans*	5%–15% Glycolytic enzymes inhibition (GEI)	Decreased ATPase, enolase, lactate dehydrogenase, protease, glucosidase, EPS and acid production activity	[196]
Purple mangosteen (*Garcinia mangostana* L.)	α-mangostin EE	*S. mutans* UA159 *S. rattus* FA-1 *S. salivarius* ATCC 13419	GEI	IC_50_ 31 µM Lactic dehydrogenase, 45 µM Aldolase, 95 µM Glyceraldehyde-3-phosphate dehydrogenase inhibition	[190]

Abbreviations: A; Anthocyanin, EE; Ethanolic Extract, EGCG; Epigallocatechingallate, EM; Extraction Method, EPS; Exopolysaccharide, FLAV; Flavonol, F-ATPase; F-Adenosine triphosphatase, GEI; Glycolytic Enzymes Inhibition, HE; Hexane Extract, IC50; Inhibition Concentration 50%, MW; Molecular Weight, PAC; Proanthocyanidin, PP; Polyphenol, Ref.; References.

**Table 7:** Inhibitory effects of phytochemicals on glucosyltransferases, aggregation and quorum sensing.

Plant/Fruit Name	Bioactive Compounds and EM	Bacterial Strain	Concentration and Assay Type	Results	Ref.
Whortleberry or Bilberry (*Vaccinium myrtillus* L.)	Molecular size of fractions; F1 <10 kDa, F2 10–100 kDa, F3 >100 kDa CJ	CI of *S. mutans* IH 113728 *A. naeslundii* AHP 28639, AHP 28651 *F. nucleatum* AHN 23952, AHN 23937	48 mg/g of SS Inhibition of aggregation and reversal activity	F2 of bilberry juice 100%	[194]
Neem (*Azadirachta indica* A. Juss.)	AE	*S. sobrinus* ATCC 27607 *S. mutans* ATCC 25175 *S. cricetus* ATCC 19642 *S. sanguis* H7PR3	250 µg/mL Bacterial aggregation	Microscopically observable bacterial aggregation	[197]
Red Wine Grape (*Vitis Vinifera* L.), and its pomace	Gallic acid Catechin Epicatechin Procyanidin B1 Procyanidin B2 Resveratrol	*S. mutans* UA159	62.5 µg/mL Inhibition of GTF B and C activities	70%–85%	[195]
Green tea and black tea (*Camellia sinensis* (L.) Kuntze), and polyphenol mixtures	Theaflavin: its mono- and digallates (+)-catechin (−)-epicatechin and their enantiomers Epigallocatechin (−)-gallocatechin HAE	*S. mutans* OMZ 176	Theaflavin 1–10 mM Inhibition of GTF activities	significant inhibition	[193]
Leaves of Oolong tea (*Camellia sinensis* (L.) Kuntze)	Oolong tea polyphenol OTF6 (polymeric polyphenol) EE	*S. mutans* MT8148R	60–850 µg/mL rGTFs (rGTFB, rGTFD, rGTFC) synthesis inhibition	50%	[197]
Rock cinquefoil (*Drymocallis rupestris* (L.) Sojak)	PRU2 PRU TAC 155 mg/g TPC 4.6 mg/g TFC 10.2 mg/g	*S. mutans* CAPM 6067 *S. sobrinus* CAPM 6070, DSM 20381, *downei* CCUG 21020 *S. sanguis* ATCC 10556	0.75–1.5 mg/mL PRU and PRU2 Inhibition of GTF activities	60%	[198]
Apple (*Malus domestica* Borkh.)	Apple condensed tannins (ACT) Apple PP and apple juice	*S. mutans* MT 8148 (serotype C) *S. sobrinus* 6715 (serotype G)	1.5–5 μg/mL ACT Inhibition of GTF activities	50%	[80]
Hop (*Humulus lupulus* L.)	High MW PP 36,000–40,000 AEE	*S. mutans* MT 8148 (serotype C) *S. sobrinus* ATCC 33478 (serotype G)	0.1% Inhibition of GTF activities	significant effect	[199]
Cranberry (*Vaccinium macrocarpon* Aiton)	FLAV A PAC	*S. mutans* UA159	PAC; 500 µg/mL FLAV; 125 µg/mL A; 200 µg/mL Inhibition of GTF B and C activities	FLAV, PAC or in combination 30%–60%	[84]
Cranberry (*Vaccinium macrocarpon* Aiton)	High MW non-dialysable material (NDM) CJ	*S. sobrinus* 6715	2 mg/mL Inhibition of GTF, FTF activities, 1 h incubation	GTF 20% FTF 40%	[115]
Cranberry (*Vaccinium macrocarpon* Aiton)	Low MW PP	*S. mutans* UA 159	500 µM/L Reduction of glucan synthesis by GTFB, GTFC	Quercetin-3-arabinofuranoside + procyanidin A2 45%	[191]
Beard lichen (*Usnea longissima* Ach.)	Herbo-metallic preparations	*S. mutans*	5%–15% Inhibition of violacein production	Partial QS inhibition	[196]
Indian gooseberry (*Emblica Officinalis* L.)	Crude and EF	*S. mutans* MTCC 497		QS inhibition (suppression of *comDE*), glucan synthesis reduction	[173]
Marupá (*Eleutherine americana* Merr.) Rose myrtle (*Rhodomyrtus tomentosa* (Aiton) Hassk.	CE of different extractive solvents	CI of *S. pyogenes* and NPRC109	250 mg/mL QS inhibition	Partial to strong inhibition; *R. tomentosa*	[90]

Abbreviations: A; Anthocyanin, ACT; Apple condensed tannins, AE; Aqueous Extract, AEE; Aqueous Ethanolic Extract, CE; Crude Extract, CEE; Crude Ethanolic Extract, CI; Clinical Isolate, CJ; Concentrated Juice, comDE; two-component signal transduction system, EE; Ethanolic Extract, EF; Ethanolic Fractions, EM; Extraction Method, FLAV; Flavonol, FTF; Fructosyltransferase, GTF; Glucosyltransferases, HAE; Hot Aqueous Extract, KDa; Kilodalton, MW; Molecular Weight, NDM; High Molecular Weight Non-Dialysable Materials Extracted From Cranberry Juice, PAC; Proanthocyanidin, PP; Polyphenol, PRU; Aqueous Extract Sub-Fraction, PRU2; Diethyl Ether Sub-Fraction, QS; Quorum Sensing, Ref.; References, SS; Solid Soluble, TFC; Total Flavonoid Content, TPC; Total Proanthocyanidins Content, TTC; Total Tannin Content.

**Figure 3:**
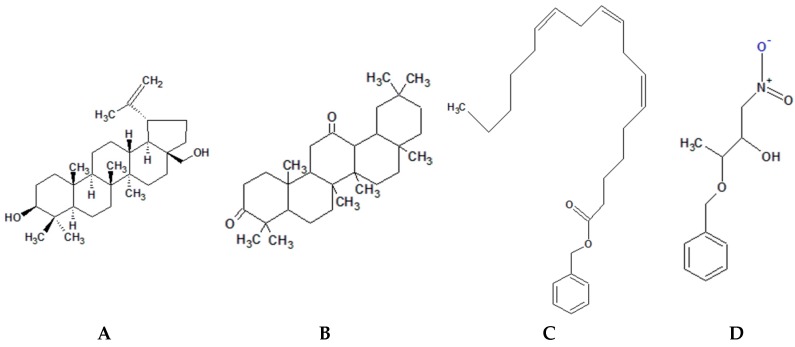
Chemical structure of phytochemicals with *S. mutans* quorum sensing inhibition activity. (**A**): Betulin; (**B**): Oleanane-3,12-dione; (**C**): Benzyl (6*Z*,9*Z*,12*Z*)-6,9,12-octadecatrienoate; (**D**): 3-Benzyloxy-1-nitrobutan-2-ol.

Crude extract of *Eleutherine americana* at 250 mg/mL inhibited the quorum-sensing of a clinical isolate of *S. pyogenes*, partially, while at the same concentration *Rhodomyrtus tomentosa* had a stronger inhibition activity [90]. Betulin, oleanane-3,12-dione, benzyl (6*Z*,9*Z*,12*Z*)-6,9,12-octadecatrienoate, and 3-benzyloxy-1-nitrobutan-2-ol possess great anti-quorum sensing inhibition activities (Figure 3A,B). Few bioactives compounds of *A. aspera* have shown to effectively interact with quorum sensing response regulators of *S. mutans* thus preventing expression of virulence elements [171]. Molecular docking revealed that *A. aspera* bioactive compounds, 3,12-oleandione and betulin, could inhibit quorum sensing by interacting with *S. mutans* OmpR subfamily QS regulatory DNA-binding response regulator and *S. mutans* glycosyltransferase (EPS synthesizing enzyme), respectively [171]. Al-Sohaibani *et al.* performed similar analysis on the bioactive compounds of *Salvadora persica* methanolic extract [172]. Results suggest that benzyl (6*Z*,9*Z*,12*Z*)-6,9,12-octadecatrienoate and 3-benzyloxy-1-nitrobutan-2-ol (Figure 3C,D) are capable of interacting with *S. mutans* OmpR subfamily QS regulatory DNA-binding response regulator thus hindering biofilm formation by this or similar quorum sensing pathway [172].

## 3. Conclusions and Prospects

Each class of classical antibacterial agents (antibiotics) usually targets different sites and processes of pathogenic bacteria. Major antimicrobial actions include disruption of membrane structure, inhibition of protein synthesis, and inhibition of production of folate coenzymes, nucleic acids, and peptidoglycans. Natural antimicrobials like their synthetic counterparts (antibiotics) target different molecules and processes to inhibit the colonization and viability of the bacteria or to inactivate bacterial toxins and or modulate the molecules and processes pre-requisite for bacteria’s metabolic pathways or reduce the rate of protein synthesis. It is worth noting that natural antimicrobial products not necessarily have to be bactericidal to suppress such processes and activities. It is plausible that a compound is likely to be efficient bacterial growth inhibitor if it can deteriorate the cytoplasmic pH, increase the permeability of plasma membrane, prevent extracellular and intracellular microbial enzyme production, interrupt bacterial metabolic pathways, or disrupt plaque and biofilm formation. As observed, there is considerable amount of scientific evidence that phytochemicals exert significant multiple anti-streptococcal effects and apart from their bactericidal effects, their main bacteriostatic strategy is the anti-adhesiveness attribute.

The efficacy of natural products as antimicrobials with fewer or no side effects is likely to depend on the structure of the compound that interacts with the toxin or pathogen and not with molecules of the host meaning that their effect is specific. This approach has become the rationale for natural drug design studies as a new field of research. Attempts have been made to understand certain features relating to phytochemical structure and the associated antibacterial activity. High molecular weight and complex phytochemicals exert greater inhibitory effects such as pentamer polyphenolic fraction of cocoa, high molecular weight non-dialyzable material of cranberry and F2 or F3 fractions of crowberry and bilberry. The side effects of the current antimicrobials and the spread of drug-resistant microorganisms have become a significant concern and a threat to successful therapy of microbial diseases. Therefore, there is an urgent demand for the discovery of safe natural compounds with diverse chemical structures and mechanisms of action satisfying both the consumer and the healthcare providers as potential useful therapeutic tools of the post-antibiotic era. Intensive research on such plants could lead to the incorporation of the most potent chemically defined extracts into nutraceuticals or natural health products and becoming a solution to this global concern of evolution of drug-resistant microorganisms.

## References

[1] Toit M.D., Huch M., Cho G.S., Franz C.M., Holzapfel W.H., Wood B.J.B. (2014). The family streptococcaceae. Lactic Acid Bacteria: Biodiversity and Taxonomy.

[2] Shi E. (2009). Flesh-Eating Bacteria: Various Strains and Virulence. Personal communication.

[3] Hayes C.S., Williamson H. (2001). Management of group a beta-hemolytic streptococcal pharyngitis. Am. Fam. Phys..

[4] Baron S., Davis C.P., Baron S. (1996). Normal flora. Medical Microbiology.

[5] Mitchell T.J. (2003). The pathogenesis of streptococcal infections: From tooth decay to meningitis. Nat. Rev. Microbiol..

[6] Nobbs A.H., Lamont R.J., Jenkinson H.F. (2009). Streptococcus adherence and colonization. Microbiol. Mol. Biol. Rev..

[7] Patterson M.J., Baron S. (1996). Streptococcus. Medical Microbiology.

[8] Facklam R.F., Martin D.R., Marguerite L., Dwight R.J., Efstratiou A., Thompson T.A., Gowan S., Kriz P., Tyrrell G.J., Kaplan E. (2002). Extension of the lancefield classification for group a streptococci by addition of 22 new m protein gene sequence types from clinical isolates: Emm103 to emm124. Clin. Infect. Dis..

[9] Public Health Agency of Canada. http://www.Phac-aspc.Gc.Ca/lab-bio/res/psds-ftss/streptococcus-agalactiae-eng.Php#footnote2.

[10] Zapun A., Vernet T., Pinho M.G. (2008). The different shapes of cocci. FEMS Microbiol. Rev..

[11] Petersen P.E., Bourgeois D., Ogawa H., Estupinan-Day S., Ndiaye C. (2005). The global burden of oral diseases and risks to oral health. Bull. World Health Organ..

[12] Matsui R., Cvitkovitch D. (2010). Acid tolerance mechanisms utilized by *Streptococcus mutans*. Future Microbiol..

[13] Stevens D.L. (1995). Streptococcal toxic-shock syndrome: Spectrum of disease, pathogenesis, and new concepts in treatment. Emerg. Infect. Dis..

[14] Carapetis J.R., Steer A.C., Mulholland E.K., Weber M. (2005). The global burden of group a streptococcal diseases. Lancet Infect. Dis..

[15] Torralba K.D., Quismorio F.P. (2009). Soft tissue infections. Rheum. Dis. Clin. N. Am..

[16] Guarner J., Sumner J., Paddock C.D., Shieh W.-J., Greer P.W., Reagan S., Fischer M., van Beneden C.A., Zaki S.R. (2006). Diagnosis of invasive group a streptococcal infections by using immunohistochemical and molecular assays. Am. J. Clin. Pathol..

[17] Sherris J.C., Ray C.G., Sherris J.C. (1990). An Introduction to Infectious Diseases. Medical Microbiology.

[18] Public Health Agency of Canada. http://www.Phac-aspc.Gc.Ca/lab-bio/res/psds-ftss/streptococcus-agalactiae-eng.Php.

[19] Kothari N.J., Morin C.A., Glennen A., Jackson D., Harper J., Schrag S.J., Lynfield R. (2009). Invasive group b streptococcal disease in the elderly, Minnesota, USA, 2003–2007. Emerg. Infect. Dis..

[20] WHO (2007). Pneumococcal conjugate vaccine for childhood immunization. Wkly. Epidemiol. Rec..

[21] O’Brien K.L., Wolfson L.J., Watt J.P., Henkle E., Deloria-Knoll M., McCall N., Lee E., Mulholland K., Levine O.S., Cherian T. (2009). Burden of disease caused by *Streptococcus pneumoniae* in children younger than 5 years: Global estimates. Lancet.

[22] Martens P., Worm S.W., Lundgren B., Konradsen H.B., Benfield T. (2004). Serotype-specific mortality from invasive *Streptococcus pneumoniae* disease revisited. BMC Infect. Dis..

[23] Huang S.S., Johnson K.M., Ray G.T., Wroe P., Lieu T.A., Moore M.R., Zell E.R., Linder J.A., Grijalva C.G., Metlay J.P. (2011). Healthcare utilization and cost of pneumococcal disease in the united states. Vaccine.

[24] Bisno A., Brito M., Collins C. (2003). Molecular basis of group a streptococcal virulence. Lancet Infect. Dis..

[25] Cunningham M.W. (2000). Pathogenesis of group a streptococcal infections. Clin. Microbiol. Rev..

[26] Ferretti J.J., McShan W.M., Ajdic D., Savic D.J., Savic G., Lyon K., Primeaux C., Sezate S., Suvorov A.N., Kenton S. (2001). Complete genome sequence of an M1 strain of *Streptococcus pyogenes*. Proc. Natl. Acad. Sci. USA.

[27] Frieden T. (2013). Antibiotic Resistance Threats in the United States.

[28] Dajani A., Taubert K., Ferrieri P., Peter G., Shulman S. (1995). Treatment of acute streptococcal pharyngitis and prevention of rheumatic fever: A statement for health professionals. Pediatrics.

[29] Kreikemeyer B., McIver K.S., Podbielski A. (2003). Virulence factor regulation and regulatory networks in *Streptococcus pyogenes* and their impact on pathogen–host interactions. Trends Microbiol..

[30] Nizet V., Rubens C.E. (2000). Pathogenic mechanisms and virulence factors of group b streptococci. Gram-Positive Pathogens.

[31] Jedrzejas M.J. (2001). Pneumococcal virulence factors: Structure and function. Microbiol. Mol. Biol. Rev..

[32] Schrag S.J., Zywicki S., Farley M.M., Reingold A.L., Harrison L.H., Lefkowitz L.B., Hadler J.L., Danila R., Cieslak P.R., Schuchat A. (2000). Group b streptococcal disease in the era of intrapartum antibiotic prophylaxis. N. Engl. J. Med..

[33] Aljicevic M., Karcic E., Bektas S., Karcic B. (2015). Representation of *Streptococcus pneumoniae* in outpatient population of sarajevo canton. Med. Arch..

[34] Moscoso M., García E., López R. (2006). Biofilm formation by *Streptococcus pneumoniae*: Role of choline, extracellular DNA, and capsular polysaccharide in microbial accretion. J. Bacteriol..

[35] Hasty D., Ofek I., Courtney H., Doyle R. (1992). Multiple adhesins of streptococci. Infect. Immun..

[36] Hall-Stoodley L., Costerton J.W., Stoodley P. (2004). Bacterial biofilms: From the natural environment to infectious diseases. Nat. Rev. Microbiol..

[37] Akiyama H., Morizane S., Yamasaki O., Oono T., Iwatsuki K. (2003). Assessment of *Streptococcus pyogenes* microcolony formation in infected skin by confocal laser scanning microscopy. J. Dermatol. Sci..

[38] Davey M.E., O’toole G.A. (2000). Microbial biofilms: From ecology to molecular genetics. Microbiol. Mol. Biol. Rev..

[39] Lemon K., Earl A., Vlamakis H., Aguilar C., Kolter R. (2008). Biofilm development with an emphasis on bacillus subtilis. Bacterial Biofilms.

[40] O’Toole G.A., Kolter R. (1998). Initiation of biofilm formation in pseudomonas fluorescens wcs365 proceeds via multiple, convergent signalling pathways: A genetic analysis. Mol. Microbiol..

[41] Cvitkovitch D.G., Li Y.H., Ellen R.P. (2003). Quorum sensing and biofilm formation in streptococcal infections. J. Clin. Investig..

[42] Fux C., Costerton J., Stewart P., Stoodley P. (2005). Survival strategies of infectious biofilms. Trends Microbiol..

[43] Götz F. (2002). Staphylococcus and biofilms. Mol. Microbiol..

[44] Navarre W.W., Schneewind O. (1999). Surface proteins of gram-positive bacteria and mechanisms of their targeting to the cell wall envelope. Microbiol. Mol. Biol. Rev..

[45] Lewis K. (2007). Persister cells, dormancy and infectious disease. Nat. Rev. Microbiol..

[46] Domenech M., García E., Moscoso M. (2012). Biofilm formation in *Streptococcus pneumoniae*. Microb. Biotechnol..

[47] Lewis K., Romeo T. (2008). Multidrug tolerance of biofilms and persister cells. Bacterial Biofilms.

[48] Chen L., Wen Y.-M. (2011). The role of bacterial biofilm in persistent infections and control strategies. Int. J. Oral Sci..

[49] Gibbons R.J. (1989). Bacterial adhesion to oral tissues: A model for infectious diseases. J. Dent. Res..

[50] Whittaker C.J., Klier C.M., Kolenbrander P.E. (1996). Mechanisms of adhesion by oral bacteria. Ann. Rev. Microbiol..

[51] Starr C.R., Engleberg N.C. (2006). Role of hyaluronidase in subcutaneous spread and growth of group a streptococcus. Infect. Immun..

[52] Fischetti V.A. (1989). Streptococcal M protein: Molecular design and biological behavior. Clin. Microbiol. Rev..

[53] Manetti A.G., Zingaretti C., Falugi F., Capo S., Bombaci M., Bagnoli F., Gambellini G., Bensi G., Mora M., Edwards A.M. (2007). *Streptococcus pyogenes* pili promote pharyngeal cell adhesion and biofilm formation. Mol. Microbiol..

[54] Lachica R., Zink D. (1984). Plasmid-associated cell surface charge and hydrophobicity of yersinia enterocolitica. Infect. Immun..

[55] Tylewska S., Hjerten S., Wadström T. (1981). Effect of subinhibitory concentrations of antibiotics on the adhesion of *Streptococcus pyogenes* to pharyngeal epithelial cells. Antimicrob. Agents Chemother..

[56] Miörner H., Johansson G., Kronvall G. (1983). Lipoteichoic acid is the major cell wall component responsible for surface hydrophobicity of group a streptococci. Infect. Immun..

[57] Westergren G., Olsson J. (1983). Hydrophobicity and adherence of oral streptococci after repeated subculture *in vitro*. Infect. Immun..

[58] Wibawan I.W.T., Lämmler C., Pasaribu F.H. (1992). Role of hydrophobic surface proteins in mediating adherence of group b streptococci to epithelial cells. J. Gen. Microbiol..

[59] Wadström T., Schmidt K.H., Kühnemund O., Havlícek J., Köhler W. (1984). Comparative studies on surface hydrophobicity of streptococcal strains of groups a, b, c, d and g. J. Gen. Microbiol..

[60] Doyle R.J. (2000). Contribution of the hydrophobic effect to microbial infection. Microbes Infect..

[61] Cotter P.D., Hill C. (2003). Surviving the acid test: Responses of gram-positive bacteria to low ph. Microbiol. Mol. Biol. Rev..

[62] Kuhnert W.L., Quivey R.G. (2003). Genetic and biochemical characterization of the F-ATPase operon from *Streptococcus sanguis* 10904. J. Bacteriol..

[63] Martinez A.R., Abranches J., Kajfasz J.K., Lemos J.A. (2010). Characterization of the *Streptococcus sobrinus* acid-stress response by interspecies microarrays and proteomics. Mol. Oral Microbiol..

[64] Ofek I., Doyle R.J. (1994). Bacterial Adhesion to Cells and Tissues.

[65] Manson M.D., Tedesco P., Berg H.C., Harold F.M., van der Drift C. (1977). A protonmotive force drives bacterial flagella. Proc. Natl. Acad. Sci. USA.

[66] Kakinuma Y. (1998). Inorganic cation transport and energy transduction in enterococcus hirae and other streptococci. Microbiol. Mol. Biol. Rev..

[67] Futai M., Noumi T., Maeda M. (1989). Atp synthase (H^+^-ATPase): Results by combined biochemical and molecular biological approaches. Ann. Rev. Biochem..

[68] Bender G.R., Sutton S.V., Marquis R.E. (1986). Acid tolerance, proton permeabilities, and membrane atpases of oral streptococci. Infect. Immun..

[69] Vadeboncoeur C., Pelletier M. (1997). The phosphoenolpyruvate: Sugar phosphotransferase system of oral streptococci and its role in the control of sugar metabolism. FEMS Microbiol. Rev..

[70] Denda K., Konishi J., Hajiro K., Oshima T., Date T., Yoshida M. (1990). Structure of an atpase operon of an acidothermophilic archaebacterium, sulfolobus acidocaldarius. J. Biol. Chem..

[71] Quivey R.G., Kuhnert W.L., Hahn K. (2001). Genetics of acid adaptation in oral streptococci. Crit. Rev. Oral Biol. Med..

[72] Kuramitsu H.K. (1993). Virulence factors of mutans streptococci: Role of molecular genetics. Crit. Rev. Oral Biol. Med..

[73] Yamashita Y., Bowen W.H., Burne R.A., Kuramitsu H.K. (1993). Role of the *Streptococcus mutans* gtf genes in caries induction in the specific-pathogen-free rat model. Infect. Immun..

[74] Bowen W.H. (2002). Do we need to be concerned about dental caries in the coming millennium?. Crit. Rev. Oral Biol. Med..

[75] Schilling K.M., Bowen W.H. (1992). Glucans synthesized *in situ* in experimental salivary pellicle function as specific binding sites for *Streptococcus mutans*. Infect. Immun..

[76] Sturr M.G., Marquis R.E. (1992). Comparative acid tolerances and inhibitor sensitivities of isolated f-atpases of oral lactic acid bacteria. Appl. Environ. Microbiol..

[77] Quivey R.G., Faustoferri R., Monahan K., Marquis R. (2000). Shifts in membrane fatty acid profiles associated with acid adaptation of *Streptococcus mutans*. FEMS Microbiol. Lett..

[78] Pedersen P.L., Amzel L.M. (1993). Atp synthases. Structure, reaction center, mechanism, and regulation of one of nature’s most unique machines. J. Biol. Chem..

[79] Higuchi M., Yamamoto Y., Poole L.B., Shimada M., Sato Y., Takahashi N., Kamio Y. (1999). Functions of two types of nadh oxidases in energy metabolism and oxidative stress of *Streptococcus mutans*. J. Bacteriol..

[80] Yanagida A., Kanda T., Tanabe M., Matsudaira F., Oliveira Cordeiro J.G. (2000). Inhibitory effects of apple polyphenols and related compounds on cariogenic factors of mutans streptococci. J. Agric. Food Chem..

[81] Matsumoto M., Hamada S., Ooshima T. (2003). Molecular analysis of the inhibitory effects of oolong tea polyphenols on glucan-binding domain of recombinant glucosyltransferases from *Streptococcus mutans* mt8148. FEMS Microbiol. Lett..

[82] Hanada N., Kuramitsu H.K. (1989). Isolation and characterization of the *Streptococcus mutans* gtfd gene, coding for primer-dependent soluble glucan synthesis. Infect. Immun..

[83] Bowen W., Koo H. (2011). Biology of *Streptococcus mutans*-derived glucosyltransferases: Role in extracellular matrix formation of cariogenic biofilms. Caries Res..

[84] Duarte S., Gregoire S., Singh A.P., Vorsa N., Schaich K., Bowen W.H., Koo H. (2006). Inhibitory effects of cranberry polyphenols on formation and acidogenicity of *Streptococcus mutans* biofilms. FEMS Microbiol. Lett..

[85] Suntharalingam P., Cvitkovitchemail D.G. (2005). Quorum sensing in streptococcal biofilm formation. Trends Microbiol..

[86] Jiang S.M., Cieslewicz M.J., Kasper D.L., Wessels M.R. (2005). Regulation of virulence by a two-component system in group b streptococcus. J. Bacteriol..

[87] Jimenez J.C., Federle M.J. (2014). Quorum sensing in group a streptococcus. Front. Cell. Infect. Microbiol..

[88] Cook L.C., LaSarre B., Federle M.J. (2013). Interspecies communication among commensal and pathogenic streptococci. MBio.

[89] Donlan R.M. (2002). Biofilms: Microbial life on surfaces. Emerg. Infect. Dis..

[90] Limsuwan S., Voravuthikunchai S.P. (2008). Boesenbergia pandurata (ROXB.) schltr., eleutherine americana merr. And rhodomyrtus tomentosa (aiton) hassk. As antibiofilm producing and antiquorum sensing in *Streptococcus pyogenes*. FEMS Immunol. Med. Microbiol..

[91] Nazzaro F., Fratianni F., Coppola R. (2013). Quorum sensing and phytochemicals. Int. J. Mol. Sci..

[92] Kaur G., Rajesh S., Princy S.A. (2015). Plausible drug targets in the *Streptococcus mutans* quorum sensing pathways to combat dental biofilms and associated risks. Indian J. Microbiol..

[93] Li Y.H., Tian X. (2012). Quorum sensing and bacterial social interactions in biofilms. Sensors.

[94] Marcy S.M. (2007). Treatment options for streptococcal pharyngitis. Clin. Pediatr..

[95] Stevens D.L., Bisno A.L., Chambers H.F., Dellinger E.P., Goldstein E.J., Gorbach S.L., Hirschmann J.V., Kaplan S.L., Montoya J.G., Wade J.C. (2014). Practice guidelines for the diagnosis and management of skin and soft tissue infections: 2014 update by the infectious diseases society of america. Clin. Infect. Dis..

[96] Ciofu O., Giwercman B., HØIBY N., Pedersen S.S. (1994). Development of antibiotic resistance in pseudomonas aeruginosa during two decades of antipseudomonal treatment at the danish cf center. Apmis.

[97] Benzie I.F.F., Wachtel-Galor S. (2011). Herbal Medicine: Biomolecular and Clinical Aspects.

[98] Savoia D. (2012). Plant-derived antimicrobial compounds: Alternatives to antibiotics. Future Microbiol..

[99] Clair S. (2010). Arnica: A proven first aid remedy for injuries and accidents. J. N. Zeal. Assoc. Med. Herbal..

[100] Shang X., Pan H., Li M., Miao X., Ding H. (2011). Lonicera japonica thunb.: Ethnopharmacology, phytochemistry and pharmacology of an important traditional chinese medicine. J. Ethnopharmacol..

[101] Yarnell E., Abascal K., Hooper C.G. (2003). Chronic sinusitis. Altern. Complement. Ther..

[102] Mathieu G., Meissa D. (2008). Traditional leafy vegetables in senegal: Diversity and medicinal uses. Afr. J. Tradit. Complement. Altern. Med..

[103] Konan N.A., Bacchi E.M., Lincopan N., Varela S.D., Varanda E.A. (2007). Acute, subacute toxicity and genotoxic effect of a hydroethanolic extract of the cashew (*Anacardium occidentale* L.). J. Ethnopharmacol..

[104] Li Y., Ooi L.S., Wang H., But P.P., Ooi V.E. (2004). Antiviral activities of medicinal herbs traditionally used in southern mainland china. Phytother. Res..

[105] Smullen J., Koutsou G., Foster H., Zumbé A., Storey D. (2007). The antibacterial activity of plant extracts containing polyphenols against *Streptococcus mutans*. Caries Res..

[106] Berahou A., Auhmani A., Fdil N., Benharref A., Jana M., Gadhi C. (2007). Antibacterial activity of quercus ilex bark’s extracts. J. Ethnopharmacol..

[107] Fabio A., Cermelli C., Fabio G., Nicoletti P., Quaglio P. (2007). Screening of the antibacterial effects of a variety of essential oils on microorganisms responsible for respiratory infections. PTR Phytother. Res..

[108] Ordoñez A., Gomez J., Cudmani N., Vattuone M., Isla M. (2003). Antimicrobial activity of nine extracts of sechium edule (jacq.) swartz. Microb. Ecol. Health Dis..

[109] Nair R., Chanda S. (2006). Activity of some medicinal plants against certain pathogenic bacterial strains. Indian J. Pharmacol..

[110] Das M., Dhanabalan R., Doss A. (2009). *In vitro* antibacterial activity of two medicinal plants against bovine udder isolated bacterial pathogens from dairy herds. Ethnobot. Leafl..

[111] Kamble M.T., Gallardo W., Yakuitiyage A., Chavan B.R., Rusydi R., Rahma A. (2014). Antimicrobial activity of bioactive herbal extracts against *Streptococcus agalactiae* biotype 2. Int. J. Basis Appl. Biol..

[112] Toivanen M., Huttunen S., Duricová J., Soininen P., Laatikainen R., Loimaranta V., Haataja S., Finne J., Lapinjoki S., Tikkanen-Kaukanen C. (2010). Screening of binding activity of *Streptococcus pneumoniae*, *Streptococcus agalactiae* and *Streptococcus suis* to berries and juices. PTR Phytother. Res..

[113] Nguelefack E.M., Ngu K.B., Atchade A., Dimo T., Tsabang N., Mbafor J.T. (2006). Phytochemical composition and *in vitro* effects of the ethyl acetate bark extract of distemonanthus benthamianus baillon (caesalpiniaceae) on *Staphylococcus aureus* and *Streptococcus agalactiae*. Cameroon J. Exp. Biol..

[114] Karmakar U.K., Biswas S.K., Chowdhury A., Raihan S.Z., Akbar M.A., Muhit M.A., Mowla R. (2012). Phytochemical investigation and evaluation of antibacterial and antioxidant potentials of asparagus racemosus. Int. J. Pharmacol..

[115] Steinberg D., Feldman M., Ofek I., Weiss E.I. (2004). Effect of a high-molecular-weight component of cranberry on constituents of dental biofilm. J. Antimicrob. Chemother..

[116] Nostro A., Cannatelli M.A., Crisafi G., Musolino A.D., Procopio F., Alonzo V. (2004). Modifications of hydrophobicity, *in vitro* adherence and cellular aggregation of *Streptococcus mutans* by *Helichrysum italicum* extract. LAM Lett. Appl. Microbiol..

[117] Yamanaka A., Kimizuka R., Kato T., Okuda K. (2004). Inhibitory effects of cranberry juice on attachment of oral streptococci and biofilm formation. Oral Microbiol. Immunol..

[118] Khare C.P., Khare C.P. (2008). Indian Medicinal Plants: An Illustrated Dictionary.

[119] Cavero R., Calvo M. (2014). Medicinal plants used for respiratory affections in navarra and their pharmacological validation. J. Ethnopharmacol..

[120] Cornu C., Joseph P., Gaillard S., Bauer C., Vedrinne C., Bissery A., Melot G., Bossard N., Belon P., Lehot J.J. (2010). No effect of a homoeopathic combination of *Arnica montana* and *Bryonia alba* on bleeding, inflammation, and ischaemia after aortic valve surgery. Br. J. Clin. Pharmacol..

[121] Ashu Agbor M., Naidoo S. (2015). Ethnomedicinal plants used by traditional healers to treat oral health problems in cameroon. Evid. Based Complement. Altern. Med..

[122] Monte A., Kabir M., Cook J., Rott M., Schwan W., Defoe L. (2006). Anti-Infective Agents and Methods of Use. U.S. Patents.

[123] Lim T.K. (2012). Parmentiera cereifera. Edible Medicinal and Non-Medicinal Plants.

[124] Locher C., Burch M., Mower H., Berestecky J., Davis H., van Poel B., Lasure A., Berghe D.V., Vlietinck A. (1995). Anti-microbial activity and anti-complement activity of extracts obtained from selected hawaiian medicinal plants. J. Ethnopharmacol..

[125] Omojasola P., Awe S. (2004). The antibacterial activity of the leaf extracts of anacardium occidentale and gossypium hirsutum against some selected microorganisms. Biosci. Res. Commun..

[126] James O., Godwin E.U., Otini I.G. (2013). *In vivo* neutralization of naja nigricollis venom by uvaria chamae. Am. J. Biochem. Biotechnol..

[127] Ogbulie J., Ogueke C., Nwanebu F. (2007). Antibacterial properties of uvaria chamae, congronema latifolium, garcinia kola, vemonia amygdalina and aframomium melegueta. Afr. J. Biotechnol..

[128] Wickens G.E., Kunkel G. (1979). The uses of the baobab (*Adansonia digitata* L.) in Africa. Taxonomic Aspects of African Economic Botany.

[129] Hebbar S., Harsha V., Shripathi V., Hegde G. (2004). Ethnomedicine of dharwad district in karnataka, india—plants used in oral health care. J. Ethnopharmacol..

[130] Sezik E., Yeşilada E., Honda G., Takaishi Y., Takeda Y., Tanaka T. (2001). Traditional medicine in turkey x. Folk medicine in central anatolia. J. Ethnopharmacol..

[131] Sezik E., Yeşİlada E., Tabata M., Honda G., Takaishi Y., Fujita T., Tanaka T., Takeda Y. (1997). Traditional medicine in turkey viii. Folk medicine in east anatolia; erzurum, erzíncan, ağri, kars, iğdir provinces. Econ. Bot..

[132] Akinpelu D.A. (2001). Antimicrobial activity of anacardium occidentale bark. Fitoterapia.

[133] Morton J.F., Dowling C.F., Morton J. (1987). In Fruits of Warm Climates.

[134] Ganesan S. (2008). Traditional oral care medicinal plants survey of tamil nadu. Nat. Prod. Rad..

[135] Okwu D.E., Iroabuchi F. (2009). Phytochemical composition and biological activities of uvaria chamae and clerodendoron splendens. J. Chem..

[136] Mollik M.A.H., Hossan M.S., Paul A.K., Taufiq-Ur-Rahman M., Jahan R., Rahmatullah M. (2010). A comparative analysis of medicinal plants used by folk medicinal healers in three districts of bangladesh and inquiry as to mode of selection of medicinal plants. Ethnobot. Res. Appl..

[137] Zhu S., Li W., Li J., Jundoria A., Sama A.E., Wang H. (2012). It is not just folklore: The aqueous extract of mung bean coat is protective against sepsis. Evid. Based Complement. Altern. Med..

[138] Chao W.W., Kuo Y.H., Hsieh S.L., Lin B.F. (2011). Inhibitory effects of ethyl acetate extract of andrographis paniculata on nf-κb trans-activation activity and lps-induced acute inflammation in mice. Evid. Based Complement. Altern. Med..

[139] Mohanasundari C., Natarajan D., Srinivasan K., Umamaheswari S., Ramachandran A. (2007). Antibacterial properties of *Passiflora foetida* L.—A common exotic medicinal plant. Afr. J. Biotechnol..

[140] Geyid A., Abebe D., Debella A., Makonnen Z., Aberra F., Teka F., Kebede T., Urga K., Yersaw K., Biza T. (2005). Screening of some medicinal plants of ethiopia for their anti-microbial properties and chemical profiles. J. Ethnopharmacol..

[141] Lim T.K. (2012). Crescentia cujete. Edible Medicinal and Non-Medicinal Plants.

[142] Akoachere J.T., Ndip R., Chenwi E., Ndip L., Njock T., Anong D. (2002). Antibacterial effects of zingiber officinale and garcinia kola on respiratory tract pathogens. East Afr. Med. J..

[143] Farrukh U., Shareef H., Mahmud S., Ali S.A., Rizwani G.H. (2008). Antibacterial activities of coccinia grandis l. Pak. J. Bot..

[144] Salari M., Amine G., Shirazi M., Hafezi R., Mohammadypour M. (2006). Antibacterial effects of eucalyptus globulus leaf extract on pathogenic bacteria isolated from specimens of patients with respiratory tract disorders. Clin. Microbiol. Infect..

[145] Rashid F., Ahmed R., Mahmood A., Ahmad Z., Bibi N., Kazmi S.U. (2007). Flavonoid glycosides fromprunus armeniaca and the antibacterial activity of a crude extract. Arch. Pharmacal Res..

[146] Cichewicz R.H., Thorpe P.A. (1996). The antimicrobial properties of chile peppers (capsicum species) and their uses in mayan medicine. J. Ethnopharmacol..

[147] Prachayasittikul S., Suphapong S., Worachartcheewan A., Lawung R., Ruchirawat S., Prachayasittikul V. (2009). Bioactive metabolites from spilanthes acmella murr. Molecules.

[148] Antonio A.G., Moraes R.S., Perrone D., Maia L.C., Santos K.R.N., Iório N.L., Farah A. (2010). Species, roasting degree and decaffeination influence the antibacterial activity of coffee against *Streptococcus mutans*. Food Chem..

[149] Antonio A., Iorio N., Pierro V., Candreva M., Farah A., Dos-Santos K., Maia L. (2011). Inhibitory properties of coffea canephora extract against oral bacteria and its effect on demineralisation of deciduous teeth. Arch. Oral Biol..

[150] Vermani A. (2009). Screening of quercus infectoria gall extracts as anti-bacterial agents against dental pathogens. Indian J. Dent. Res..

[151] Hwang J.K., Shim J.S., Chung J.Y. (2004). Anticariogenic activity of some tropical medicinal plants against *Streptococcus mutans*. Fitoterapia.

[152] Almeida L.S.B.D., Murata R.M., Yatsuda R., Dos Santos M.H., Nagem T.J., Alencar S.M.D., Koo H., Rosalen P.L. (2008). Antimicrobial activity of rheedia brasiliensis and 7-epiclusianone against *Streptococcus mutans*. Phytomedicine.

[153] Xu X., Zhou X.D., Wu C.D. (2011). The tea catechin epigallocatechin gallate suppresses cariogenic virulence factors of *Streptococcus mutans*. Antimicrob. Agents Chemother..

[154] Khan R., Zakir M., Afaq S.H., Latif A., Khan A.U. (2010). Activity of solvent extracts of prosopis spicigera, zingiber officinale and trachyspermum ammi against multidrug resistant bacterial and fungal strains. J. Infect. Dev. Ctries..

[155] Khan R., Adil M., Danishuddin M., Verma P.K., Khan A.U. (2012). *In vitro* and *in vivo* inhibition of *Streptococcus mutans* biofilm by trachyspermum ammi seeds: An approach of alternative medicine. Phytomedicine.

[156] Zheng Y., Liu Z., Ebersole J., Huang C.B. (2009). A new antibacterial compound from luo han kuo fruit extract (siraitia grosvenori). J. Asian Natl. Prod. Res..

[157] Mu J. (1998). Anti-cariogenicity of maceration extract of momordica grosvenori: Laboratory study. Chin. J. Stomatol..

[158] Bernardshaw S., Johnson E., Hetland G. (2005). An extract of the mushroom agaricus blazei murill administered orally protects against systemic *Streptococcus pneumoniae* infection in mice. Scand. J. Immunol..

[159] Hetland G., Samuelsen A., Loslash V., Paulsen B., Aaberge I., Groeng E.C., Michaelsen T. (2000). Protective effect of plantago major l. Pectin polysaccharide against systemic *Streptococcus pneumoniae* infection in mice. Scand. J. Immunol..

[160] Koné W.M., Atindehou K.K., Kacou-N A., Dosso M. (2007). Evaluation of 17 medicinal plants from northern cote d’ivoire for their *in vitro* activity against *Streptococcus pneumoniae*. Afr. J. Tradit. Complement. Altern. Med..

[161] Tepe B., Daferera D., Sokmen A., Sokmen M., Polissiou M. (2005). Antimicrobial and antioxidant activities of the essential oil and various extracts of salvia tomentosa miller (lamiaceae). Food Chem..

[162] Kuta F.A., Ndamitso M.M., Ibrahim H. (2008). Antibacterial effect of eucalyptus globulus leaves extract on antibiotic resistant strains of *Streptococcus pneumoniae*. Appl. Med. Inform..

[163] Kuta F., Damisa D., Adamu A., Nwoha E., Bello I. (2014). Antibacterial activity of euphorbia hirta against *Streptococcus pneumoniae*, klebsiella pneumoniae and proteus vulgaris. Bayero J. Pure Appl. Sci..

[164] Janecki A., Kolodziej H. (2010). Anti-adhesive activities of flavan-3-ols and proanthocyanidins in the interaction of group a-streptococci and human epithelial cells. Molecules.

[165] Percival R.S., Devine D.A., Duggal M.S., Chartron S., Marsh P.D. (2006). The effect of cocoa polyphenols on the growth, metabolism, and biofilm formation by *Streptococcus mutans* and *Streptococcus sanguinis*. EOS Eur. J. Oral Sci..

[166] Furiga A., Lonvaud Funel A., Dorignac G., Badet C. (2008). *In vitro* anti-bacterial and anti-adherence effects of natural polyphenolic compounds on oral bacteria. JAM J. Appl. Microbiol..

[167] Rahim Z.H.A., Khan H.B.S.G. (2006). Comparative studies on the effect of crude aqueous (ca) and solvent (cm) extracts of clove on the cariogenic properties of *Streptococcus mutans*. J. Oral Sci..

[168] Matsumoto M., Tsuji M., Okuda J., Sasaki H., Nakano K., Osawa K., Shimura S., Ooshima T. (2004). Inhibitory effects of cacao bean husk extract on plaque formation *in vitro* and *in vivo*. Eur. J. Oral Sci..

[169] Prabu G.R., Gnanamani A., Sadulla S. (2006). Guaijaverin—A plant flavonoid as potential antiplaque agent against *Streptococcus mutans*. J. Appl. Microbiol..

[170] Yamanaka-Okada A., Sato E., Kouchi T., Kimizuka R., Kato T., Okuda K. (2008). Inhibitory effect of cranberry polyphenol on cariogenic bacteria. Bull. Tokyo Dent. Coll..

[171] Murugan K., Sekar K., Sangeetha S., Ranjitha S., Sohaibani S. (2013). Antibiofilm and quorum sensing inhibitory activity of achyranthes aspera on cariogenic *Streptococcus mutans*: An *in vitro* and in silico study. Pharm. Biol..

[172] Al-Sohaibani S., Murugan K. (2012). Anti-biofilm activity of salvadora persica on cariogenic isolates of *Streptococcus mutans*: *In vitro* and molecular docking studies. Biofouling.

[173] Hasan S., Danishuddin M., Adil M., Singh K., Verma P.K., Khan A.U. (2012). Efficacy of e. Officinalis on the cariogenic properties of *Streptococcus mutans*: A novel and alternative approach to suppress quorum-sensing mechanism. PLoS ONE.

[174] Somanah J., Bourdon E., Bahorun T., Aruoma O.I. (2013). The inhibitory effect of a fermented papaya preparation on growth, hydrophobicity, and acid production of *Streptococcus mutans*, *Streptococcus mitis*, and *Lactobacillus acidophilus*: Its implications in oral health improvement of diabetics. FSN3 Food Sci. Nutr..

[175] Green A.E., Rowlands R.S., Cooper R.A., Maddocks S.E. (2012). The effect of the flavonol morin on adhesion and aggregation of *Streptococcus pyogenes*. FEMS Microbiol. Lett..

[176] Zhou L., Ding Y., Chen W., Zhang P., Chen Y., Lv X. (2013). The *in vitro* study of ursolic acid and oleanolic acid inhibiting cariogenic microorganisms as well as biofilm. ODI Oral Dis..

[177] Evans J., Gregory R.L., Huang R. (2015). Polyphenol effect on *Streptococcus mutans* comc-biofilm. Indiana Univ. Sch. Dent..

[178] Kang M.S., Oh J.S., Kang I.C., Hong S.J., Choi C.H. (2008). Inhibitory effect of methyl gallate and gallic acid on oral bacteria. J. Microbiol..

[179] Sendamangalam V., Choi O.K., Kim D., Seo Y. (2011). The anti-biofouling effect of polyphenols against *Streptococcus mutans*. Biofouling.

[180] Wang Y., Chung F.F., Lee S.M., Dykes G.A. (2013). Inhibition of attachment of oral bacteria to immortalized human gingival fibroblasts (HGF-1) by tea extracts and tea components. BMC Res. Notes.

[181] Xiao Y., Liu T., Zhan L., Zhou X. (2000). The effects of tea polyphenols on the adherence of cariogenic bacterium to the collagen *in vitro*. West China J. Stomatol..

[182] Feng G., Klein M.I., Gregoire S., Singh A.P., Vorsa N., Koo H. (2013). The specific degree-of-polymerization of a-type proanthocyanidin oligomers impacts *Streptococcus mutans* glucan-mediated adhesion and transcriptome responses within biofilms. Biofouling.

[183] Janecki A., Conrad A., Engels I., Frank U., Kolodziej H. (2011). Evaluation of an aqueous-ethanolic extract from pelargonium sidoides (EPs^®^ 7630) for its activity against group A-streptococci adhesion to human HEp-2 epithelial cells. J. Ethnopharmacol..

[184] Stauder M., Papetti A., Mascherpa D., Schito A.M., Gazzani G., Pruzzo C., Daglia M. (2010). Antiadhesion and antibiofilm activities of high molecular weight coffee components against *Streptococcus mutans*. J. Agric. Food Chem..

[185] Koo H., Nino de Guzman P., Schobel B.D., Vacca Smith A.V., Bowen W.H. (2006). Influence of cranberry juice on glucan-mediated processes involved in *Streptococcus mutans* biofilm development. Caries Res..

[186] Zheng J., Ramirez V.D. (2000). Inhibition of mitochondrial proton F0F1-ATPase/ATP synthase by polyphenolic phytochemicals. Br. J. Pharmacol..

[187] McKenna E., Smith J.S., Coll K.E., Mazack E.K., Mayer E.J., Antanavage J., Wiedmann R.T., Johnson R.G. (1996). Dissociation of phospholamban regulation of cardiac sarcoplasmic reticulum Ca^2+^ ATPase by quercetin. J. Biol. Chem..

[188] Hirano T., Oka K., Akiba M. (1989). Effects of synthetic and naturally occurring flavonoids on Na^+^, K^+^-atpase: Aspects of the structure-activity relationship and action mechanism. Life Sci..

[189] Lang D.R., Racker E. (1974). Effects of quercetin and F1 inhibitor on mitochondrial atpase and energy-linked reactions in submitochondrial particles. Biochim. Biophys. Acta.

[190] Nguyen P.T.M., Marquis R.E. (2011). Antimicrobial actions of α-mangostin against oral streptococci. Can. J. Microbiol..

[191] Gregoire S., Singh A., Vorsa N., Koo H. (2007). Influence of cranberry phenolics on glucan synthesis by glucosyltransferases and *Streptococcus mutans* acidogenicity. J. Appl. Microbiol..

[192] Adamson G.E., Lazarus S.A., Mitchell A.E., Prior R.L., Cao G., Jacobs P.H., Kremers B.G., Hammerstone J.F., Rucker R.B., Ritter K.A. (1999). HPLC method for the quantification of procyanidins in cocoa and chocolate samples and correlation to total antioxidant capacity. J. Agric. Food Chem..

[193] Hattori M., Kusumoto I.T., Namba T., Ishigami T., Hara Y. (1990). Effect of tea polyphenols on glucan synthesis by glucosyltransferase from *Streptococcus mutans*. Chem. Pharm. Bull..

[194] Riihinen K., Ryynanen A., Toivanen M., Kononen E., Torronen R., Tikkanen-Kaukanen C. (2011). Antiaggregation potential of berry fractions against pairs of *Streptococcus mutans* with fusobacterium nucleatum or actinomyces naeslundii. Phytother. Res. PTR.

[195] Thimothe J., Bonsi I.A., Padilla-Zakour O.I., Koo H. (2007). Chemical characterization of red wine grape (vitis vinifera and vitis interspecific hybrids) and pomace phenolic extracts and their biological activity against *Streptococcus mutans*. J. Agric. Food Chem..

[196] Singh B.N., Pandey G., Jadaun V., Singh S., Bajpai R., Nayaka S., Naqvi A.H., Rawat A.K.S., Upreti D.K., Singh B.R. (2015). Development and characterization of a novel swarna-based herbo-metallic colloidal nano-formulation–inhibitor of *Streptococcus mutans* quorum sensing. RSC Adv..

[197] Wolinsky L.E., Mania S., Nachnani S., Ling S. (1996). The inhibiting effect of aqueous azadirachta indica (neem) extract upon bacterial properties influencing *in vitro* plaque formation. J. Dent. Res..

[198] Tomczyk M., Pleszczyńska M., Wiater A. (2012). *In vitro* anticariogenic effects of aerial parts of drymocallis rupestris and its phytochemical profile. Planta Med..

[199] Tagashira M., Uchiyama K., Yoshimura T., Shirota M., Uemitsu N. (1997). Inhibition by hop bract polyphenols of cellular adherence and water-insoluble glucan synthesis of mutans streptococci. Biosci. Biotechnol. Biochem..

